# Genomic and Transcriptomic Analyses Revealed DdSTE2 Play a Role in Constricting Ring Formation in the Nematode-Trapping Fungi *Drechslerella dactyloides*

**DOI:** 10.3390/microorganisms12112190

**Published:** 2024-10-30

**Authors:** Cheng-Lin Wu, Ren-Qiao Wang, Jin-Ting Yang, Jia-Mei Sun, Yan-Rui Xu, Jianping Xu, Ke-Qin Zhang, Lian-Ming Liang

**Affiliations:** 1 State Key Laboratory for Conservation and Utilization of Bio-Resources in Yunnan, Yunnan University, Kunming 650500, China; wu799579894@163.com (C.-L.W.); 15368118669@163.com (R.-Q.W.); jintingyang2022@163.com (J.-T.Y.); sunjiamei0064@163.com (J.-M.S.); 18388511258@163.com (Y.-R.X.); 2 Department of Biology, McMaster University, Hamilton, ON L8S 4K1, Canada; jpxu@mcmaster.ca

**Keywords:** carnivorous fungus, *Drechslerella dactyloides*, constricting ring, trap formation, mutagenesis, STE2

## Abstract

The carnivorous fungus *Drechslerella dactyloides* can form constricting rings through hyphal specialization to capture nematodes. The formation of constricting rings is a prerequisite for capturing nematodes and a characteristic of entering the carnivorous stage. Currently, there is limited research on the molecular mechanism of constricting ring formation. In this study, two *D. dactyloides* mutants unable to form constricting rings were obtained through UV irradiation mutagenesis, and their growth and development phenotypes were compared with the wild-type strain. Transcriptome comparisons revealed differences between the mutants and the wild-type strain in metabolic pathways related to cell wall structure, peroxisomes, lipid metabolism, and MAPK signal transduction, which we validated through qPCR. We further deleted one differentially expressed gene, *DdSTE2,* of the MAPK pathway and confirmed its role in conidiogenesis and trap formation in *D. dactyloides*. Together, our results indicate that the remodeling of cell wall structure, peroxisomes, lipid metabolism, and MAPK signal transduction pathways are involved in the formation and maturation of *D. dactyloides* constricting rings. We discuss the implications of these results for utilizing these fungi to control animal and plant parasitic nematodes.

## 1. Introduction

Nematode-trapping fungi (NTF) are a group of fungi capable of preying on various nematodes [1]. These fungi have two living states: saprophytic and predatory. In nutrient-rich environments, they survive in a saprophytic state, while in environments with low nutrients or nitrogen levels, predatory nematode-trapping fungi can form various traps such as three-dimensional networks, adhesive knobs, adhesive columns, constriction or non-constricting rings, etc., to capture nematodes through adhesion or mechanical force [2,3]. Studying nematode-trapping fungi contributes to the biocontrol of animal- or plant-parasitic nematodes [4].

Unlike other extensively studied nematode-trapping fungi, *Drechslerella dactyloides* (M. Scholler, Hagedorn and A. Rubner, 1999; Kingdom: Fungi, Phylum: Ascomycota, Class: Orbiliomycetes, Order: Orbiliales, Family: Orbiliaceae, Genus: Drechslerella) is a particularly unique fungus capable of producing constricting rings. In addition, while other nematode-trapping fungi require inducers, such as ascarosides, to produce traps, *D. dactyloides* can spontaneously produce traps without external inducers [5,6]. A constricting ring is composed of three ring cells; when a nematode enters the constricting ring and stimulates its inner wall, three ring cells rapidly inflate and capturing the nematode through mechanical force generated by cell expansion [7,8,9]. The production and inflation of the ring cells are crucial for capturing nematodes, but there is currently little understanding of the mechanisms underlying trap formation and constricting ring inflation in *D. dactyloides*.

To investigate the molecular mechanisms underlying the formation of constricting rings, it is necessary to study the process at the genetic level. Proteins such as Crz1 and STE12 are important in the Ca^2+^/calcineurin-dependent signaling pathway and the MAPK pathway. Knocking out the genes encoding DdaCrz1 and DdaSTE12 in *D. dactyloides* led to a reduction in the formation of constricting rings, demonstrating that the Ca^2+^/calmodulin-dependent signaling pathway and MAPK pathway are involved in the ring formation in *D. dactyloides* [10,11]. t-SNARE and v-SNARE are involved in the fusion of intracellular and extracellular vesicles and cell membranes. Knocking out the genes encoding v-SNARE proteins DdSnc1 and DdVam7 revealed that constricting ring cells cannot form large vesicles. It was also determined that the fusion of small vesicles to form large vesicles and the formation of Palisade-shaped Membrane-building Structures (PMS) within the ring cells are critical for constricting ring inflation [12,13].

Mutants that completely fail to produce constricting rings or form abnormal constricting rings can be compared for mutations and differentially expressed genes. Such comparisons can help reveal a range of genes involved in regulating constricting ring formation. This strategy has been used in other species to select genes involved in regulating specific phenotypes. For example, UV mutagenesis in the entomopathogenic fungus *Beauveria bassiana* (Bals.-Criv.; Kingdom: Fungi, Phylum: Ascomycota, Class: Sordariomycetes, Order: Hypocreales, Family: Cordycipitaceae, Genus: Beauveria) led to the identification of pathways related to its virulence [14]. Analysis of natural mutations in *Candida albicans* (C.P. Robin; Kingdom: Fungi, Phylum: Ascomycota, Class: Pichiomycetes, Order: Serinales, Family: Debaryomycetaceae, Genus: Candida) identified genomic factors associated with specific phenotypes [15]. Exploration of the paclitaxel biosynthetic pathway in *Aspergillus aculeatinus* (Noonim, Frisvad, Varga and Samson, 2008; Kingdom: Fungi, Phylum: Ascomycota, Class: Eurotiomycetes, Order: Eurotiales, Family: Aspergillaceae, Genus: Aspergillus) was achieved through mutagenesis to increase production of the drug paclitaxel [16]. Using Ethyl methanesulfonate and UV as mutagens, Huang et al. screened mutants with defects in trap morphogenesis in the nematode-trapping fungus *Arthrobotrys oligospora* (Baral and E. Weber, 2020; Kingdom: Fungi, Phylum: Ascomycota, Class: Orbiliomycetes, Order: Orbiliales, Fami-ly: Orbiliaceae, Genus: Arthrobotrys), and identified a candidate gene, YBP-1, which was further confirmed through targeted molecular manipulations [17].

In this study, we obtained two mutants (DW06 and DW07) of *D. dactyloides* through UV mutagenesis that failed to form constricting rings. We analyzed the genomes and transcriptomes of these two mutants, identified gene and pathways related to constricting ring formation. In addition, we selected the gene encoding DdSTE2, a homologous protein of STE2 in the MAPK pathway, for knockout in *D. dactyloides*. Our analyses proved that DdSTE2 reversely regulates conidia formation and positively regulates constricting ring trap formation in *D. dactyloides*.

## 2. Materials and Methods

### 2.1. Strains and Growth Conditions

A wild-type *D. dactyloides* strain was isolated from field soil in Yunnan Province, China (25°24′ N, 99°38′ E) and stored in the Microbial Bank of Southwest Wild Species Germplasm Resource Center in China (http://www.genobank.org/, accessed on 21 October 2024), with voucher number YMF1.00031. The wild-type strain, along with UV-induced mutants DW06 and DW07, were cultured on potato dextrose agar (PDA, 20% potato, 2% dextrose, and 2% agar, *w*/*v*), tryptone glucose agar (TG, 1% tryptone, 1% dextrose, and 2% agar, *w*/*v*), and corn meal agar (CMA, 25% corn meal, and 2% agar, *w*/*v*) at 28 °C. The nematode *Caenorhabditis elegans* (Maupas, 1900; Kingdom: Metazoa, Phylum: Nematoda, Class: Chromadorea, Order: Rhabditida, Family: Rhabditidae, Genus: Caenorhabditis) was cultured on nematode growth medium (NGM) agar plates at 22 °C and fed with Escherichia coli OP50 strain [18].

### 2.2. Ultraviolet-Irradiation and Screening of Mutants

The *D. dactyloides* strain YMF1.00031 was inoculated onto corn meal agar (CMA) and incubated at 28 °C for 10 days. Conidia were harvested by scraping with a glass rod, suspended in sterile deionized water, and filtered through six layers of filter paper to remove hyphae. Conidial concentration was adjusted to 10^6^ conidia/mL using a hemocytometer. For UV irradiation, a suspension of 10^6^ conidia/mL (200 μL) was exposed to UV light (30 W, λ = 254 nm, distance = 30 cm) for different durations (0 s, 15 s, 30 s, 1 min, and 2 min). After irradiation, 100 μL of both unirradiated and irradiated cells were spread on PDA plates at 28 °C in the dark for 24 h to test for viability. The irradiation time resulting in a death rate of 70–80% (30 s) was chosen as the UV mutagenesis condition.

Soil extract was prepared following the method of Persmark, L. [19,20]. The suspension of conidia at a concentration of 10^6 conidia/mL, irradiated under the above UV conditions for 30 s, was mixed with cultured soil suspension at a ratio of 1:20 and incubated at 22 °C for 48 h to induce. Conidia were harvested after induction, and those not forming normal constricting rings were picked under a microscope and cultured on CMA. When the colony diameter reached 6 cm, conidia were harvested using the above method and induced with soil suspension to observe the conidial constricting ring formation rate. UV mutants with a constricting ring formation rate <5% were passaged for 5 generations to ensure that selected mutants had stable phenotypes. Our screening identified UV mutants DW06 and DW07, showing no constricting ring formation over at least five culture transfers.

### 2.3. Colonial Morphology, Growth Rate, Conidial Yield, and Germination Rate of Wild-Type and UV-Induced Mutant Strains

After culturing the wild-type and two UV-induced mutants on the PDA medium for 10 days, we created 8 mm diameter holes at the edges of the colonies and inoculated them onto PDA, tryptone glucose agar (TG), and corn meal agar (CMA) media. These plates were incubated at 28 °C for 10 days, and their colony diameters were measured every 2 days. Conidia were harvested and counted, and their morphology was observed. Finally, 1000 conidia of the wild-type and two UV-induced mutant strains were spread on 6 cm PDA plates and incubated for 24 h to estimate their conidial germination rates.

### 2.4. Analysis of Hyphal Growth of Wild-Type and UV-Induced Mutants Under Different Stresses

After 10 days of cultivation on the PDA medium, 8 mm diameter disks were cut at the edges of the colonies of the WT and two UV-induced mutants. These disks were inoculated onto the PDA medium supplemented with various inhibitors to test the Relative Growth Inhibition (RGI) of the wild-type and two UV-induced mutants. Different concentrations of NaCl (0.1 M, 0.2 M, 0.3 M) were added to test resistance to osmotic stress; different concentrations of SDS (0.01%, 0.02%, 0.03%) were added to test resistance to detergent; different concentrations of Menadione (0.03 mM, 0.06 mM) were added to test resistance to oxidative stress. Menadione was chosen instead of H_2_O_2_ because the wild-type *D. dactyloides* strain is highly sensitive to H_2_O_2_ [11].

### 2.5. Constricting Ring Formation Introduction of Wild-Type and UV-Induced Strains

The wild-type strain and the two mutants were cultured on the CMA medium for 10 days. From these plates, approximately 1000 conidia were harvested from each strain and inoculated onto 2% water agar (WA) plates (6 cm) and incubated at 28 °C for 3 days. Subsequently, approximately 1000 *C. elegans* worms were added to each plate. After 24 h of induction, the number of constricting rings formed by the wild-type and the two mutants was counted.

### 2.6. Genomic DNA Library Preparation and Sequencing

The hyphae of WT and two mutants were harvested, and their genomic DNA was extracted using the CTAB method. The quality and quantity of extracted DNA were monitored on 1% agarose gel and by ND-2000 (NanoDrop Technologies, Wilmington, DE, USA). Only high-quality DNA sample (OD260/280 = 1.8~2.0, OD260/230 < 2.0) was used to construct the DNA sequencing library. A total of 0.5 μg DNA per sample was used as input material for DNA sequencing library preparations. The sequencing library was generated using Truseq Nano DNA HT Sample Prep Kit (Illumina, San Diego, CA, USA) following the manufacturer’s recommendations, and index codes were added to each sample. Briefly, the genomic DNA sample was fragmented by sonication to a size of ~350 bp. Then, DNA fragments were end-polished, poly (A)-tail added, and ligated with the full-length adapter for Illumina sequencing, followed by further a PCR amplification. After PCR products were purified (AMPure XP system), libraries were analyzed for size distribution by Agilent 2100 Bioanalyzer and quantified by real-time PCR (3nM). The paired-end library was sequenced with the Illumina NovaSeq System at Shanghai Majorbio Bio-pharm Technology Co., Ltd. (Shanghai, China).

### 2.7. Variant Discovery

Raw reads of low quality (mean phred score < 20), including reads containing adapter contamination and unrecognizable nucleotide (N base > 10), were trimmed or discarded by the software Fastp v0.20.0 [21]. Reads after trimming were mapped to the reference genome [*D. dactyloides* YMF1.00031, Yunnan University (https://www.ncbi.nlm.nih.gov/datasets/genome/GCA_028984885.1/, accessed on 21 October 2024)] using BWA-MEME software v1.0.6 under default mapping parameters [22].

Following the modified GATK Best practices, the alignment bam files were sorted by samtools and PCR duplicates marked by MarkDuplicated [23,24]. After base quality recalibration, genomic variants were called for SNPs (Single Nucleotide Polymorphisms) and InDels (Insertion and Deletions) across all three samples using the Haplotyper and Gvcftyper programs [25].

Variants were then filtered using standard hard filtering parameters following the GATK Best Practices pipeline. SNPs and InDels were categorized based on their positions on the chromosome, including intergenic regions, exons, introns, splicing sites, untranslated regions, and 1 kb upstream and downstream regions, and on their effects, including missense, start codon gain or loss, stop codon gain or loss and splicing mutations.

Raw sequencing data generated in this project are available in the Sequence Read Archive (SRA) with the BioProject accession number PRJNA1110844 (https://www.ncbi.nlm.nih.gov/sra/PRJNA1110844, accessed on 21 October 2024).

### 2.8. Gene Ontology Enrichment of Variants

Functional enrichment analyses, including Gene Ontology (GO) and Kyoto Encyclopedia of Genes and Genomes (KEGG), were conducted for the genomic variants that were different between the two UV-induced mutants and the wild-type strain. Enrichment analyses were carried out using the R package clusterProfiler v3.19 [26].

### 2.9. Sample Preparation and RNA-Sequencing

The mycelia induced by *C. elegans* for 30 h from the WT and two UV-induced mutant strains were harvested. Total RNA was extracted from the mycelia using TRIzol^®^ reagent (Invitrogen, Carlsbad, CA, USA) according to the manufacturer’s instructions, with 5 replicates for each sample. DNase I (TaKaRa, Dalian, China) was used to remove genomic DNA. Subsequently, the RNA quality was measured using a 5300 Bioanalyzer (Agilent Technologies, Palo Alto, CA, USA) and quantified using an ND-2000 spectrophotometer (NanoDrop Technologies). Only high-quality RNA samples (OD260/280 = 1.8~2.2, OD260/230 ≥ 2.0, RQN ≥ 6.5, 28S:18S ≥ 1.0, >1 μg) were selected for library construction.

RNA purification, reverse transcription, library construction, and sequencing were performed by Shanghai Majorbio Bio-pharm Technology Co., Ltd. (Shanghai, China). For RNA-seq transcriptome library preparation, the Illumina^®^ Stranded mRNA Prep Ligation kit (San Diego, CA, USA) was used with a total RNA concentration of 1 μg. Messenger RNA was isolated using the polyA selection method with oligo (dT) beads, followed by fragmentation. Subsequently, double-stranded cDNA was synthesized using the SuperScript Double-Stranded cDNA Synthesis kit (Invitrogen, Carlsbad, CA, USA) and random hexamer primers. The synthesized cDNA was then subjected to end repair, phosphorylation, and adapter ligation according to the library construction protocol. The cDNA library, with target fragments of 300 bp, was selected on a 2% Low Range Ultra agarose gel, followed by 15 cycles of PCR amplification using Phusion DNA polymerase (NEB, Ipswich, MA, USA). After quantification using Qubit 4.0, the sequencing libraries were sequenced on the NovaSeq X Plus platform (PE150) using the NovaSeq Reagent Kit.

### 2.10. Processing and Analysis of RNA-Sequencing Data

The raw paired-end reads were trimmed and quality controlled using fastp with default parameters [21]. Subsequently, clean reads were mapped to the reference genome in directional mode using HISAT2 [27]. StringTie was employed to assemble the mapped reads for each sample [28]. RSEM was utilized to quantify gene abundance [29]. Essentially, differential expression analysis was performed using DESeq2 version 1.24.0 [30]. Genes with |log2FC| ≥ 1 and FDR < 0.05 were considered as differentially expressed genes (DEG) with significant expression differences. Additionally, functional enrichment analysis, including GO and KEGG, was conducted to identify which DEGs were significantly enriched in GO terms and metabolic pathways compared to the whole transcriptome background, with Bonferroni-corrected *p*-values <0.05. GO functional enrichment and KEGG pathway analyses were performed separately using Goatools v0.6.5 and Python script software, respectively.

Raw transcriptome data are available in the Sequence Read Archive (SRA) with the BioProject accession number PRJNA1110844 (https://www.ncbi.nlm.nih.gov/sra/PRJNA1110844, accessed on 21 October 2024).

### 2.11. Quantitative PCR Verification

The mycelia from the wild-type (WT) strain without nematode induction or induced with nematodes for 30 h (WT + 30 h) were collected as qPCR samples. Total RNA was extracted using the RaPure Total RNA Micro Kit (Guangzhou Magen Biotechnology Co., Ltd., Guangzhou, China). cDNA was synthesized using the PrimeScriptTM RT reagent Kit with gDNA Eraser (Perfect Real Time) (TaKaRa, Kusatsu, Japan). Real-time PCR was performed using the LightCycler 480 SYBR green I master mix (Applied Biosystems, Darmstadt, Germany). The PCR conditions were as follows: 95 °C for 5 min, followed by 45 cycles of 95 °C for 10 s, 58 °C for 10 s, and 72 °C for 20 s. The β-tubulin gene was utilized as an internal standard. The primer sequences used for qPCR are listed in Table S1. The relative transcript level of each gene was calculated using the threshold cycle (2^−ΔΔCT^) method.

### 2.12. Gene Disruption of DdSTE2

Disruption of the DdSTE12-encoding gene (Dda_7765, GeneBank: KAJ6257975.1) in *D. dactyloides* was achieved by homologous recombination [31]. The 5′ and 3′ flanking sequences, approximately 2.5 kb on each side of the *DdSTE2* open reading frame (ORF), were used. The 5′ flanking sequence was amplified with primers DdSTE2-5F-F and DdSTE2-5F-R, and the 3′ flanking sequence was amplified with primers DdSTE2-3F-F and DdSTE2-3F-R (Table S2). The 5′ and 3′ flanking sequences were cloned to the 5′ and 3′ ends of the hygromycin resistance gene box of the pAg1-H3 vector, respectively, and the plasmid was amplified with primers DdSTE2-5F-F and DdSTE2-3F-R to obtain the 5′ flanking sequence of DdSTE2-hygromycin resistance gene box-the 3′ flanking sequence of *DdSTE2*. Finally, protoplasts were prepared, and the amplified gene fragment was delivered to achieve the disruption of the *DdSTE2* gene. Positive transformants were verified by PCR using primers DdSTE2bt-F and DdSTE2bt-R inside the gene and primers DdSTE2QC-F and DdSTE2QC-R outside the gene (Table S2). The tubulin gene fragment of 508 bp was used as an internal control with primers Tubulin-F and Tubulin-R.

### 2.13. Colonial Morphology, Growth Rate, Conidial Yield and Germination Rate of Wild-Type Strain and ∆DdSTE2 Mutants

The growth morphology of WT and ∆*DdSTE2* mutants on the PDA medium was observed. After culturing WT and ∆*DdSTE2* mutants on the PDA medium for 10 days, create 8 mm diameter holes at the edges of the colonies and inoculate them onto PDA and CMA media. The plates were incubated at 28 °C for 10 days, and the colony diameters were measured every 2 days. The conidia were recovered with sterilized deionized water, and the conidia morphology was observed and counted. 100 conidia of WT and ∆*DdSTE2* mutants were coated on 6 cm PDA plates for 24 h, and the conidia germination rate was counted.

### 2.14. Hyphal Constricting Ring Formation of Wild-Type and ∆DdSTE2 Mutants After Introducing Nematodes

To compare the ability of constricting rings, approximately 500 conidia were harvested from wild-type and ∆*DdSTE2* mutants cultured in CMA media for 10 days, seeded on 2% WA plates, and incubated at 28 °C for 3 days. Then, about 500 *C. elegans* were added to each dish. After 24 h, the number of constricting rings formed by wild-type and ∆*DdSTE2* mutants was counted.

## 3. Results

### 3.1. Two Mutant Strains That Lose the Ability to Form Traps Are Obtained

**Figure 1:**
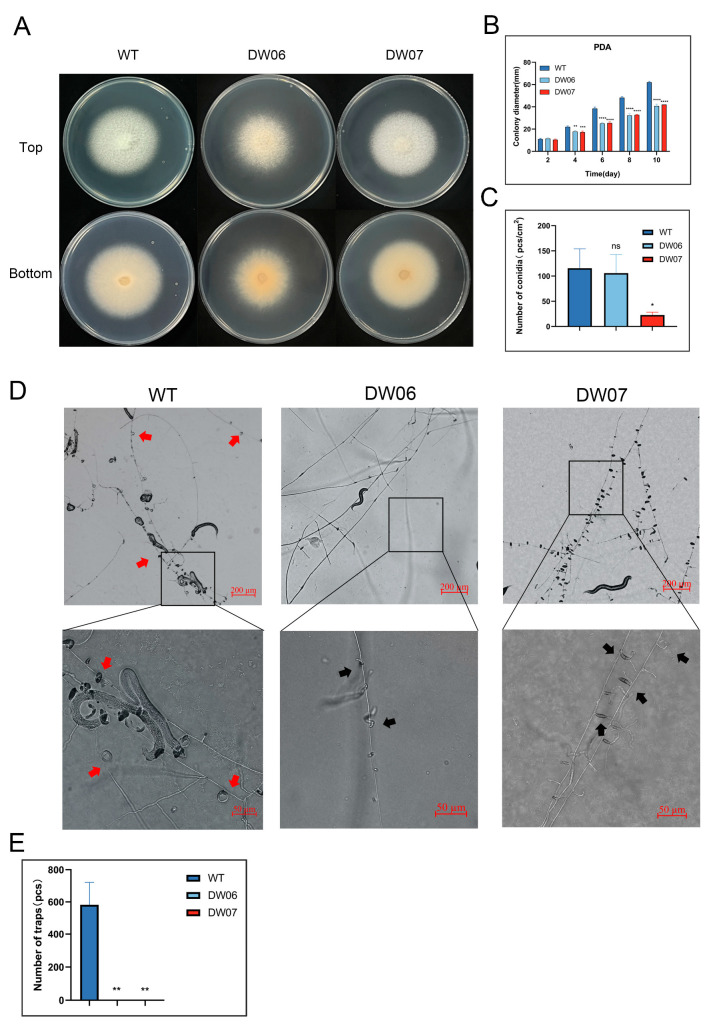
Colony morphology, growth rate, conidia production, and trap formation ability of wild-type (WT) strain and UV-induced mutants (DW06 and DW07). (**A**) Top and bottom views of colonies of WT, DW06, and DW07 on the PDA medium. (**B**) Colony diameter of WT and mutants on the PDA medium. Data represent mean ± standard deviation (SD) (*n* = 4). (**C**) Spore production per square centimeter of colonies of WT and mutants after 10 days on CMA medium. Data represent mean ± standard deviation (SD) (*n* = 3). (**D**) Traps formed by WT, DW06, and DW07 strains after co-cultivation with nematodes for 24 h. The red arrow points to the normal constricting ring trap, and the black arrow points to the abnormal constricting ring trap. (**E**) Number of normal constricting rings induced by nematodes. Data represent mean ± standard deviation (SD) (*n* = 3). In (**B**), two-way ANOVA analysis was conducted. In (**C**,**E**), *t*-test analysis was performed. *, *p* ≤ 0.05; **, *p* ≤ 0.01; ***, *p* ≤ 0.001; ****, *p* ≤ 0.0001; ns, *p* > 0.05.

Wild-type *D. dactyloides* conidia were exposed to ultraviolet radiation for 30 s, followed by screening for their ability to form constricting rings after 48 h of induction using soil extract. Two mutants unable to form rings (DW06 and DW07) were selected (Figure S1). The colonies of both wild-type and mutant DW07 were white on PDA and TG mediums, while the center of the colonies of DW06 was slightly red, but there was no morphological difference between wild-type and mutant strains on the CMA medium (Figure 1A and Figure S2A). However, the mutant strains grew significantly slower than those of the wild-type on the PDA, CMA, and TG medium (Figure 1B and Figure S2C,D). Conidia production and conidial germination ability were tested by culturing DW06, DW07, and WT on the CMA medium at 28 °C for 10 days. There was no significant difference between DW06 and the wild-type strain, but the conidia production of DW07 was significantly lower than that of both the wild-type strain and DW06 (Figure 1C). There were no significant differences in conidia germination rates among the wild-type, DW06, and DW07 (Figure S2E).

To compare the tolerance of the wild-type strain and mutants to osmotic, detergent, and oxidative stresses, the three strains were cultured on the PDA medium supplemented with NaCl, SDS, and Menadione. Experiments have shown that under NaCl and SDS stress conditions, DW06 and DW07 exhibited stronger resistance than the wild-type strain (Figure S3A–C). However, under Menadione stress, DW06 showed no significant difference compared to WT, while DW07 was more sensitive to Menadione (Figure S3A,D).

The formation of constricting rings by *D. dactyloides* is an important means for capturing nematodes to obtain nitrogen sources [7]. After inoculating 1000 conidia of each wild-type, DW06, and DW07 strains into 2% WA plates and culturing for 72 h, nematodes were added for 24 h. The results showed that many intact contractile rings were formed on the hyphae of the wild-type strain, while no structurally intact contractile rings were observed on the hyphae of DW06 and DW07 (Figure 1D,E). In addition, many abnormal constricting ring-like structures appeared on the hyphae of DW07. Together, the results demonstrated that DW06 and DW07 were unable to form normal constricting rings.

### 3.2. Mutants DW06 and DW07 Exhibit Significant Genomic Variations

To identify the UV-induced mutations, we resequenced the genomes of the two mutants, DW06 and DW07, as well as the wild-type (WT), compared them with the reference genome of *D. dactyloides* YMF1.00031, and annotated the mutation sites to infer their functions. Excluding mutations in intergenic regions, introns, and synonymous variants, whole-genome resequencing revealed, compared to the strain YMF1.00031 reference genome, there were 281, 449, and 389 SNPs in the WT, DW06 and DW07, respectively (Figure S4A, Tables S3 and S5); and 387, 394, and 385 InDels in WT, DW06, and DW07, respectively (Figure S4B, Tables S4 and S6).

**Figure 2:**
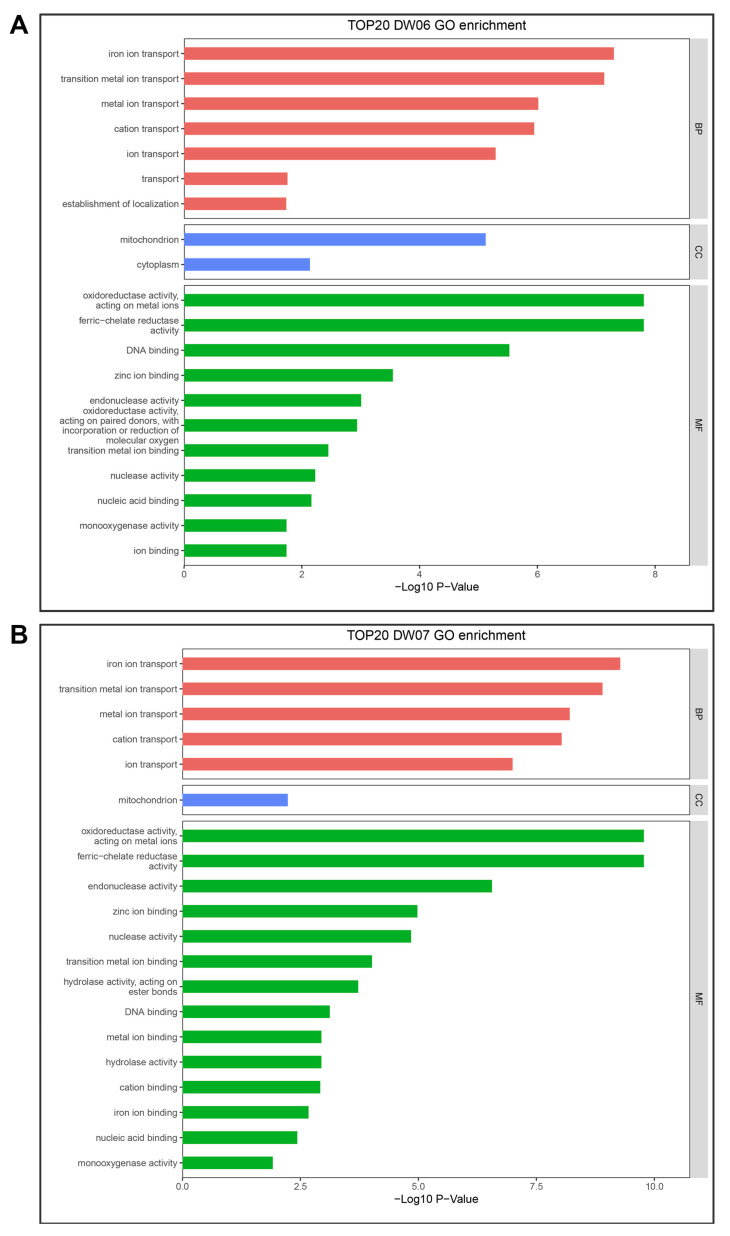
The top 20 GO terms for mutated genes in DW06 (**A**) and DW07 (**B**), two UV-induced mutation strains.

DW06 and DW07 were aligned with the wild-type gene sequences to conduct enrichment analysis of mutation sites using Gene Ontologies (GO) (Figure 2, Tables S7 and S8). The results indicated that genes related to molecular functions and biological processes were highly enriched. A large number of mutation sites were enriched in oxidoreductase activity, acting on metal ions (GO:0016722), DNA binding (GO:0003677), cation transport (GO:0006812), and iron ion transport (GO:0006826). In addition, chitinase activity (GO:0004568), oxidoreductase activity (GO:0016491), catalytic activity (GO:0003824), and integral component of membrane (GO:0016021) were enriched (Tables S7 and S8). This suggests that mutated genes are mostly associated with cellular structure, cellular metabolism, and signal transduction.

### 3.3. UV-Induced Mutants Exhibited Distinct Transcriptomes

To help elucidate the molecular mechanisms underlying the inability of UV-induced mutants to form traps, we compared the transcriptome profiles of WT and UV-induced mutants. RNA was extracted from WT, DW06, and DW07 in the presence of nematodes for 30 h, and a total of 15 libraries (five replications per strain) were constructed for RNA sequencing (Table S9). The number of clean reads ranged from 47,335,190 to 62,066,892. The Q30 values (sequencing quality > 99.9%) were all above 90%, indicating good data quality.

**Figure 3:**
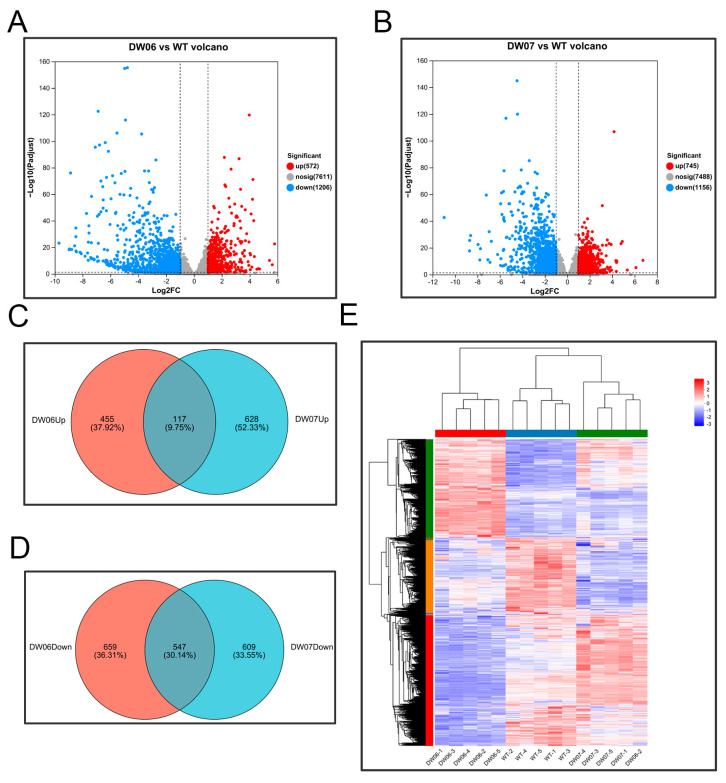
Overview of differentially expressed genes (DEGs) between two UV-induced mutants and wild-type. (**A**,**B**) Volcano plots of DEGs (P.adj < 0.05, |log2FC| ≥ 1) between DW06 vs. WT and DW07 vs. WT. (**C**,**D**) Venn diagrams of upregulated DEGs (**C**) and downregulated DEGs (**D**) in the two gene sets. The orange and blue circles represent the two genomes (DW06 vs. WT, DW07 vs. WT), respectively. (**E**) Heatmap of clustering analysis. Each column represents a sample, and each row represents a gene. The normalized gene expression values are represented in different colors.

In the gene sets of DW06 vs. WT and DW07 vs. WT, 1778 and 1901 differentially expressed genes (DEGs) were identified, respectively (Padj < 0.05, |log2FC| ≥ 1). In the DW06 vs. WT comparison, 572 genes were upregulated, and 1206 genes were downregulated in DW06 (Figure 3A). In the DW07 vs. WT comparison, 745 genes were upregulated, and 1156 genes were downregulated in DW07 (Figure 3B). Venn diagrams showed 72 (Figure 3C) and 44 (Figure 3D) shared DEGs between the two comparisons for upregulated and down-regulated, respectively. Hierarchical clustering heatmaps demonstrated that genes with similar expression patterns and samples were closely clustered together (Figure 3E).

### 3.4. Functional Annotation and Enrichment Analysis of DEGs between Wild-Type and Two UV-Induced Mutants Reveal Genes Involved in Trap Formation and Maturation

DEGs from the DW06 vs. WT and DW07 vs. WT comparisons were assigned to GO terms, and the top 30 most abundant GO terms were listed (Figure S5A). The abundance of DEGs annotated to molecular functions and cellular components indicates that *D. dactyloides* accompanies a series of signal transduction-related substances’ transport and binding when sensing the nearby existence of nematodes, forming contraction rings. DEGs from the DW06 vs. WT and DW07 vs. WT comparisons were also assigned to the Kyoto Encyclopedia of Genes and Genomes (KEGG) pathways, and the top 20 most abundant KEGG pathways were listed (Figure S5B,C). Most shared DEGs from both the DW06 vs. WT and the DW07 vs. WT comparisons were annotated to carbohydrate metabolism, amino acid metabolism, and lipid metabolism in the metabolism category.

**Figure 4:**
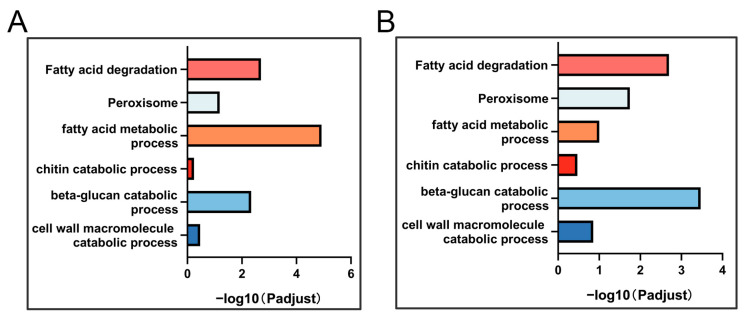
GO terms and KEGG pathways related to trap formation enriched in DW06 vs. WT gene set and DW07 vs. WT gene set. (**A**) GO terms and KEGG pathways related to trap formation enriched in DW06 vs. WT gene set. (**B**) GO terms and KEGG pathways related to trap formation enriched in DW07 vs. WT gene set.

GO and KEGG enrichment analyses were conducted on genes from the DW06 vs. WT and DW07 vs. WT gene sets. According to the analysis of GO and KEGG enrichment results, a large number of genes related to carbohydrate metabolism, lipid metabolism, and amino acid metabolism were enriched, which may lead to a significant decrease in the growth rate of the two UV-induced mutants compared with the wild-type (Figures S6 and S7, Tables S12–S15). Additionally, gene terms and pathways related to trap formation were also enriched (Figure 4). The contraction rings of *D. dactyloides* are formed by specialized hyphae, and during the process of contraction ring formation and maturation, several layers of cell wall are formed on the inner wall of the ring, which involves cell wall remodeling [8]. Enrichment of the GO term “cell wall macromolecule catabolic process (GO:0016998)” was found in both the DW06 vs. WT and the DW07 vs. WT DEG sets. The genes (Dda_0210/Dda_3377/Dda_5701/Dda_3282/Dda_7889) in this GO term were downregulated in DW06 and DW07 compared to the wild-type strain. Additionally, enrichment of the GO term beta-glucan catabolic process (GO:0051275) was observed, and the genes (Dda_8810 and Dda_0739) in this GO term were homologous to glucanase genes, showing downregulation compared to WT expression levels. The GO term chitin catabolic process (GO:0006032) was also enriched in the DEGs in both the DW06 vs. WT and the DW07 vs. WT comparisons (Dda_7450 and Dda_0366).

Most predatory nematode-trapping fungi possess dense bodies associated with peroxisomes in their trapping cells. Peroxisomes are highly involved in fatty acid metabolism, providing hyphal cells with energy for trap formation, and are associated with trap formation. Enrichment of the Peroxisome (map04146) pathway and Fatty acid degradation (map00071) pathway was found in the KEGG enrichment results of DEGs in the DW06 vs. WT and DW07 vs. WT comparisons. Enrichment of the GO term fatty acid metabolic process (GO:0006631) was also found in the GO enrichment results of both DEG sets. Differential expression of homologous protein genes involved in β-oxidation (Dda_7173/Dda_1334) and homologous protein genes involved in peroxisome-related pathways (Dda_2984) was observed compared to WT in these enrichment results.

The presence of nematodes leads to the transition of *D. dactyloides* from a saprophytic state to a predatory state, a process involving several signal cascades. Although the MAPK signaling pathway–yeast (map04011) was not significantly enriched in the KEGG enrichment analyses in the DW06 vs. WT and DW07 vs. WT comparisons, significant differences in expression were observed compared to WT for multiple proteins in this pathway, including homologous genes of STE2 (Dda_7765), WSC (Dda_7285), Mkk1_2 (Dda_8878), and Swe1 (Dda_4825) (Tables S10 and S11).

### 3.5. The qPCR Results Demonstrate That Trap Formation Is Related to Cell Wall, Peroxisomes, Lipid Metabolism, and MAPK Signaling Pathway

**Figure 5:**
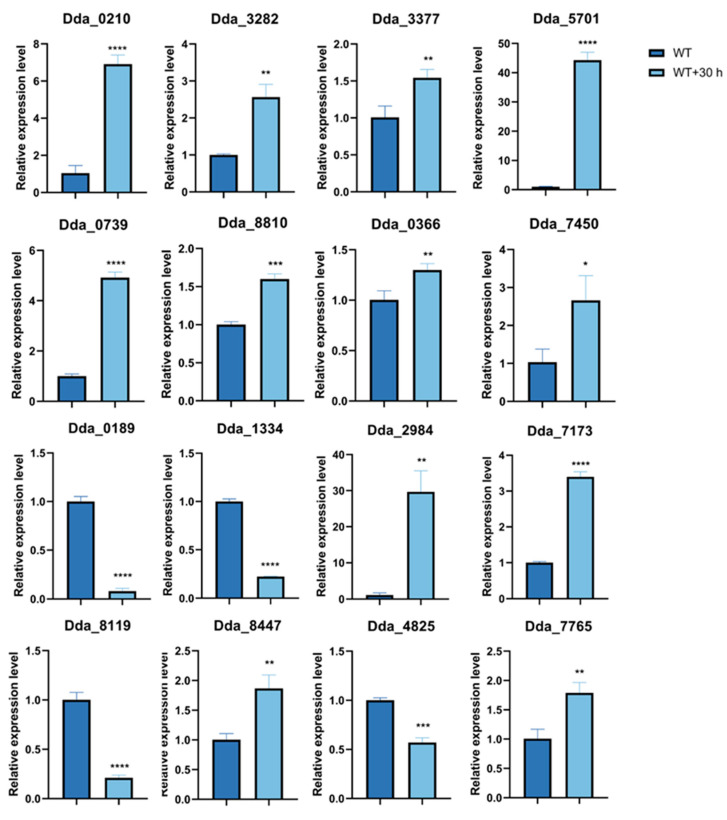
qPCR validation of genes involved in trap formation in *D. dactyloides*. Total RNA was extracted from the wild-type (WT) plants without nematode induction and the wild-type plants induced with nematodes for 30 h (WT + 30 h). Genes enriched in GO enrichment and KEGG enrichment were selected for qPCR validation. Analysis was performed using *t*-test. * *p* < 0.05; ** *p* < 0.01; *** *p* < 0.001; **** *p* < 0.0001.

To validate the transcriptome results, we selected genes that were consistently differentially expressed in the transcriptome analysis and tested the expression levels of these genes before and after the addition of nematodes to the wild-type (WT) strain through qPCR (Figure 5). According to the qPCR results, 30 h after nematode exposure, the expression of homologous protein genes associated with cell wall degradation metabolism and structure such as glycoside hydrolase (Dda_0210), Endo-1,4-beta-xylanase (Dda_3282), Endoglucanase (Dda_3377/Dda0739/Dda_8810), and glycoside hydrolase (Dda_5701) was significantly upregulated in WT, with Dda_5701 showing the most significant upregulation (over 40-fold). Additionally, the expression levels of genes associated with cell wall structure remodeling, such as chitotriosidase (Dda_7450) and endochitinase (Dda_0366), were also upregulated.

Thirty hours after nematode induction, the expression levels of genes related to peroxisomes and lipid metabolism showed significant differences compared to the wild-type, with the homologous genes of long-chain-fatty-acid-CoA ligase (Dda_2984), Acetyl-acyltransferase B (Dda_7173), and acyl-CoA dehydrogenase (Dda_8447) significantly upregulated. However, the expression levels of the homologous genes of 3-ketoacyl-CoA thiolase (Dda_1334), nonspecific lipid-transfer protein (Dda_8119), and another acyl-CoA dehydrogenase (Dda_0189) were significantly downregulated. Furthermore, significant differences were observed in the expression levels of genes related to the MAPK signaling pathway, including the homologous protein genes of STE2 (Dda_7765) and Swe1 (Dda_4825).

### 3.6. Disruption of DdSTE2 Resulted in a Significant Decrease in Trap Formation

**Figure 6:**
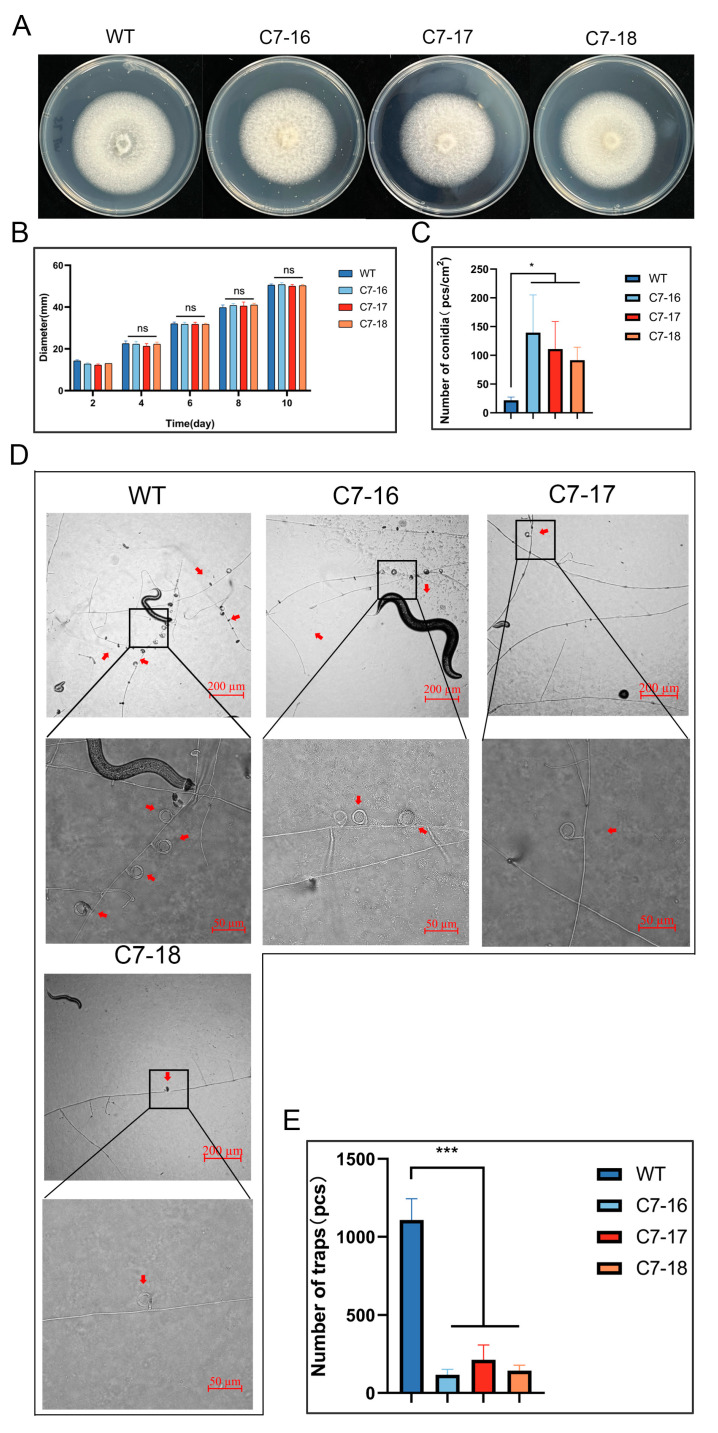
Colony morphology, growth rate, conidia production, and trap formation ability of wild-type (WT) and ∆DdSTE2 mutants (C7–16,C7–17, and C7–18). (**A**) Colony morphology of WT and mutants on the PDA medium. (**B**) Colony diameter of WT and ∆DdSTE2 mutants on the PDA medium. Data represent mean ± standard deviation (SD) (*n* = 4). (**C**) Spore production per square centimeter of colonies of WT and mutants after 10 days on CMA medium. Data represent mean ± standard deviation (SD) (*n* = 3). (**D**) Traps formed by wild-type and ∆DdSTE2 mutants after co-cultivation with nematodes for 24 h. The red arrow points to the constricting ring traps. (**E**) Number of traps induced by nematodes. Data represent mean ± standard deviation (SD) (*n* = 3). In (**B**), two-way ANOVA analysis was conducted. In (**C**,**E**), *t*-test analysis was performed. * *p* ≤ 0.05; *** *p* ≤ 0.001; ns *p* > 0.05.

Previous studies have demonstrated that the MAPK pathway is involved in the formation of traps. Here, the STE2 homologous gene DdSTE2, a member of the MAPK pathway, was selected for knockout analyses. This gene was chosen because it showed different patterns between the transcriptome analysis and the qPCR analysis. Three strains of ∆DdSTE2 mutants, C7–16, C7–17, and C7–18, were obtained by homologous recombination through PCR (Figure S8). There was no significant difference in colony morphology and growth rate between WT and ∆DdSTE2 mutants on PDA and CMA media (Figure 6A,B and Figure S9A,B). However, the DdSTE2 knockout mutants showed a significantly higher number of conidia than WT on CMA (Figure 6C). There was no significant difference in the germination ability of conidia between WT and ∆DdSTE2 mutants (Figure S9C).

500 conidia of WT and △DdSTE2 mutants were inoculated in 2% WA plates for 72 h, nematodes were added and incubated for 24 h, and the number of constricting ring traps was counted. While a large number of intact constricting rings were formed on WT hyphae, there were few intact constricting rings on the hyphae of ∆DdSTE2 mutants (Figure 6D,E). The experimental results showed that the deletion of DdSTE2 significantly affected the formation of constricting rings and reversely regulated conidia formation in *D. dactyloides*.

## 4. Discussion

Nematode-trapping fungi predating nematodes in nutrient-deficient environments is of great ecological and economic significance [32]. The constricting rings of *D. dactyloides* represent a distinct type of trap among nematode-trapping fungi, unlike others that employ adhesive knobs, adhesive nets, and branches, or non-constricting rings to adhere nematodes [33]. Upon stimulation, the cells of the constricting ring rapidly expand inward, increasing in size by threefold within 0.1 s, thereby mechanically immobilizing the nematode [34]. However, information on the molecular mechanisms underlying trap formation in *D. dactyloides* is limited, and specific genes directly regulating trap formation in *D. dactyloides* have not been identified. Using genomic and transcriptomic data, this study attempted to explore the potential genetic basis underlying constricting ring formation.

**Figure 7:**
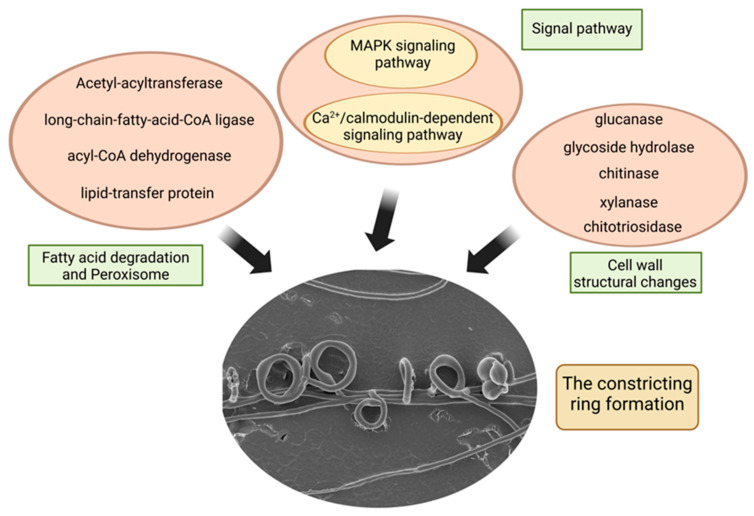
A proposed model for trap formation in *D. dactyloides*. Protein information can be found in Tables S10 and S11. Created with BioRender.com, accessed on 21 October 2024.

In this study, through UV irradiation mutagenesis, we first obtained two UV-induced mutants that were unable to form constricting rings. Overall, the mycelial growth rate of the mutants was significantly slower than that of the wild-type, but they showed stronger resistance to certain environmental stresses such as SDS and NaCl treatments. Subsequent genomic and transcriptomic analyses revealed a large number of genes enriched in carbohydrate metabolism, lipid metabolism, and amino acid metabolism, which may contribute to the significant decrease in the growth ability of these mutant strains. Furthermore, a set of genes and pathways potentially involved in trap formation were screened. The differential expression patterns of these genes and pathways were confirmed in the wild-type strain through qPCR analysis, consistent with their involvement in trap formation. The combined genomic, transcriptomic, and qPCR data and past research suggest a model of nematode trap formation in *D. dactyloides* [11,12,13] (Figure 7). In this model, Trap formation in *D. dactyloides* is involved by multiple pathways and gene sets. These processes include (i) Signal pathways are activated; (ii) Fatty acid metabolism is enhanced, providing energy for the trap formation; (iii) Cell wall structural changes to ensure that the contractile ring expands inward after the nematode enters. Finally, we performed phenotypic analyses of ∆*DdSTE2* mutants by disrupting the *STE2* homologous gene *DdSTE2* in the MAPK pathway and found that disruption of DdSTE2 resulted in a significant decrease in trap formation and increased conidia formation in *D. dactyloides*. Experimental results show that the DdSTE2 protein is essential for the trap and conidia formation in *D. dactyloides*.

The synthesis and structural changes in the cell wall are closely linked to the formation and maturation of *D. dactyloides* traps. Upon stimulation of the constricting ring, the ring cells expand three folds from their original size. In addition to requiring sufficient cell membrane, an adequate cell wall is also necessary to ensure this expansion. Previous studies have found that during trap maturation in *D. dactyloides*, a large number of vesicles accumulate on the inner wall of the ring, with DdSnc1 participating in vesicle fusion with the plasma membrane, ensuring sufficient cell membrane during ring expansion. However, even in the absence of DdSnc1 (in ∆*DdSnc1*), although vesicles do not aggregate on the inner wall of the ring, the inner cell wall of the trap still thickens, indicating that dynamic changes in the cell wall during expansion are mediated by other genes [12]. Additionally, ultrastructural studies of constricting rings revealed that the inner cell wall consisted of four or five layers, while the outer cell wall consisted of only two layers [8]. Since the cell wall is mainly composed of glucans and chitin, changes in the expression levels of glucanase-related genes and chitinase-related genes during trap formation and maturation may ensure sufficient extensibility of the cell wall during expansion. In this study, genes related to cell wall decomposition metabolism were highly upregulated, indicating their close involvement in the maturation process of the ring.

During the expansion of the constricting ring trap, a large amount of material needs to enter the ring cells to ensure sufficient mechanical force (turgor pressure) to immobilize the nematode. However, the mechanism ensuring the rapid expansion of the ring is still unclear. *Magnaporthe oryzae* (Cavara, 1892; Kingdom: Fungi, Phylum: Ascomycota, Class: Sordariomycetes, Order: Magnaporthales, Family: Pyriculariaceae, Genus: Pyricularia), a plant pathogenic fungus, achieves invasion by using appressoria to exert turgor pressure and insert infection pegs into plant cell walls, with glycerol providing the turgor pressure [8]. Transcriptomic analysis of *M. oryzae* also showed widespread upregulation of genes related to long-chain fatty acid metabolism, indicating the involvement of fatty acid degradation metabolism in appressorium formation and function [8]. Additionally, previous studies have found that lipid droplets accumulate in the vegetative hyphae of another nematode-trapping fungus, *A*. *oligospora*, during late infection, and new vegetative hyphae develop from the initial nematode-capturing fungal network when the lipid droplets disappear [8]. Therefore, lipid metabolism may provide not only abundant energy for trap formation in *D. dactyloides* but also potentially provide turgor pressure for the expansion of the constricting ring. Our results revealed that genes encoding peroxisomes and proteins related to lipid metabolism, such as acyl-CoA dehydrogenase, 3-ketoacyl-CoA thiolase, and nonspecific lipid-transfer protein, were significantly downregulated after nematode induction. Additionally, we found that some lipid metabolism-related genes were upregulated after nematode induction, a phenomenon also observed in *A. oligospora* [8]. This may indicate that certain nutrients released by nematodes are first converted into lipids by fungi. Subsequently, lipids are degraded via the β-oxidation pathway on peroxisomes to support the growth of new vegetative hyphae. Further studies involving individual gene knockouts are needed to determine the roles of these proteins in trap formation. In summary, peroxisomes and genes related to lipid metabolism play a significant role in the formation and maturation of *D. dactyloides* traps.

Signal transduction is crucial for the transition of nematode-trapping fungi from a saprophytic lifestyle to a predatory one and for trap formation [35,36,37,38]. Previous studies have found that homologous genes of a key protein STE12 in the MAPK pathway are involved in trap formation and ring expansion in *D. dactyloides*, demonstrating the involvement of the MAPK pathway in trap formation and maturation [10]. In this experiment, significant differences were also found in the expression levels of homologous genes of the important protein genes in the MAPK pathway, such as STE2 (Dda_7765) and Swe1 (Dda_4825), before and after nematode exposure. Additionally, Swe1, as a negative regulator of the cell cycle, which can inhibit CDC28 by phosphorylation to interfere with cell mitosis [39], showed decreased expression after nematode induction, indicating that the cell cycle is also closely related to trap formation.

The STE family is a class of pheromone-sensing G protein-coupled receptors, in which STE2 is involved in the sexual reproduction of fungi as a receptor for α-pheromones [40,41]. In addition, it has been shown that STE2 affects virulence by regulating the taxa-specific growth of plant pathogenic fungi to the host, and it is speculated that STE2 may be involved in the perception of the host by plant pathogenic fungi [42]. In this study, we constructed a gene knockout strain ∆*DdSTE2* of the STE2 homologous protein gene *DdSTE2* in *D. dactyloides* for the first time. Through analyzing the growth and trap production capacity of ∆*DdSTE2* mutants, we demonstrated that DdSTE2 is inversely involved in conidia formation and positively regulates trap formation in *D. dactyloides*. The absence of DdSTE2 may have led to a significant decrease in the perception of nematodes by *D. dactyloides*, causing reduced trap formation.

Research on pathogenic fungi that affect plant pests, such as nematodes and insects, can contribute to the stability of natural ecosystems and provide significant support for agricultural production [37,38,42]. In this study, the genomes and transcriptomes of two non-trapping UV-induced *D. dactyloides* mutants were analyzed, and important pathways and genes related to their pathogenicity were screened. Through our genomic and transcriptomic analyses, as well as qPCR results, we believe that the reorganization of cell wall structure, peroxisomes, lipid metabolism, and MAPK signal transduction pathways are highly involved in the formation and maturation of *D. dactyloides* traps. In addition, gene knockout proved that the STE2 homologous protein gene *DdSTE2* in the MAPK signaling pathway is indispensable for the formation of intact constricting rings formation in *D. dactyloides*. This information aids in understanding this particular predatory fungus and provides resources for future studies on the formation and maturation of *D. dactyloides* constricting rings. Additionally, it provides a foundation for comparing similarities and differences between *D. dactyloides* and other predatory nematode-trapping fungi. These data also have important implications for the practical development of improved biological control agents against nematode pests.

## Data Availability

The original contributions presented in the study are included in the article/Supplementary Material, further inquiries can be directed to the corresponding authors.

## Supplementary Materials

The following supporting information can be downloaded at: https://www.mdpi.com/article/10.3390/microorganisms12112190/s1, Table S1: Primers used in qPCR verification; Table S2: Primers used in knockout fragment construction and positive transformant validation; Table S3: statistical tables of SNP; Table S4: statistical tables of InDel; Table S5: SNP function annotations; Table S6: InDel function annotations; Table S7: DW06 GO enrichment; Table S8: DW07 GO enrichment; Table S9: RNA-sequencing of the hyphae of Wild-type strain and ultraviolet (UV)-induced mutants collected from the PDA medium; Table S10: DEGs for DW06 mutant versus WT; Table S11: DEGs for DW07mutant versus WT; Table S12: DW06 DEGs GO enrichment; Table S13: DW07 DEGs GO enrichment; Table S14: DW06 DEGs KEGG enrichment; Table S15: DW07 DEGs KEGG enrichment; Figure S1: Conidia trap-forming capacity of wild-type (WT) and UV-induced mutants (DW06 and DW07); Figure S2: Colony morphology and growth rate on TG and CMA media, and percentage of conidia germination in wild-type (WT) strain and UV-induced mutants (DW06 and DW07); Figure S3: Tolerance of wild-type (WT) and UV-induced mutants (DW06 and DW07) to osmotic, oxidative, and cell wall inhibitory stress; Figure S4: Functional annotation analysis of wild-type (WT) and UV-induced mutants (DW06 and DW07) compared to reference genomic SNPs and InDel; Figure S5: GO and KEGG annotations. (A) Top 30 enriched GO_level_2 annotations in the DW06 vs. WT and DW07 vs. WT comparison gene sets. (B) and (C) Top 20 enriched KEGG annotations in the DW06 vs WT and DW07 vs WT comparison gene sets; Figure S6: The top 50 most significant GO terms enrichment results for DW06 vs. WT gene sets and DW07 vs. WT gene sets; Figure S7: The top 30 most significant KEGG pathways enrichment results for DW06 vs. WT gene sets and DW07 vs. WT gene sets; Figure S8: Disruption strategy and verification of ΔDdaSTE12. Figure S9: Colony morphology and rowth rate on CMA medium, and percentage of conidia germination in wild-type (WT) strain and △DdSTE2 mutants (C7–16, C7–17 and C7–18).

## References

[1] Liu K., Tian J., Xiang M., Liu X. (2012). How carnivorous fungi use three-celled constricting rings to trap nematodes. Protein Cell.

[2] Barron G.L. (2003). Predatory fungi; wood decay, and the carbon cycle. Biodiversity.

[3] Schmidt A.R., Do H., Perrichot V. (2007). Carnivorous fungi from Cretaceous amber. Science.

[4] Tunlid A., Ahrén D. (2011). Molecular Mechanisms of the Interaction Between Nematode-Trapping Fungi and Nematodes: Lessons from Genomics. Biological Control of Plant-Parasitic Nematodes: Building Coherence between Microbial Ecology and Molecular Mechanisms.

[5] Choe A., von Reuss S.H., Kogan D., Gasser R.B., Platzer E.G., Schroeder F.C., Sternberg P.W. (2012). Ascaroside Signaling Is Widely Conserved among Nematodes. Curr. Biol..

[6] Hsueh Y.P., Mahanti P., Schroeder F.C., Sternberg P.W. (2013). Nematode-Trapping Fungi Eavesdrop on Nematode Pheromones. Curr. Biol..

[7] Yang Y., Yang E., An Z., Liu X. (2007). Evolution of nematode-trapping cells of predatory fungi of the Orbiliaceae based on evidence from rRNA-encoding DNA and multiprotein sequences. Proc. Natl. Acad. Sci. USA.

[8] Dowsett J.A., Reid J., Caeseele L.V. (1977). Transmission and scanning electron microscope observations on the trapping of nematode by *Dactylaria brochopaga*. Can. J. Bot..

[9] Muller H.G. (1958). The constricting ring mechanism of two predacious hyphomycetes. Trans. Br. Mycol. Soc..

[10] Fan Y., Zhang W., Chen Y., Xiang M., Liu X. (2021). DdaSTE12 is involved in trap formation, ring inflation, conidiation, and vegetative growth in the nematode-trapping fungus *Drechslerella dactyloides*. Appl. Microbiol. Biotechnol..

[11] Zhao X., Fan Y., Xiang M., Kang S., Wang S., Liu X. (2022). DdaCrz1, a C2H2-Type Transcription Factor, Regulates Growth, Conidiation, and Stress Resistance in the Nematode-Trapping Fungus *Drechslerella dactyloides*. J. Fungi.

[12] Chen Y., Liu J., Kang S., Wei D., Fan Y., Xiang M., Liu X. (2023). A palisade-shaped membrane reservoir is required for rapid ring cell inflation in *Drechslerella dactyloides*. Nat. Commun..

[13] Chen Y., Liu J., Fan Y., Xiang M., Kang S., Wei D., Liu X. (2022). SNARE Protein DdVam7 of the Nematode-Trapping Fungus *Drechslerella dactyloides* Regulates Vegetative Growth, Conidiation, and the Predatory Process via Vacuole Assembly. Microbiol. Spectr..

[14] Sun Y.X., Zhang B.X., Zhang W.T., Wang Q., Toufeeq S., Rao X.J. (2023). UV-induced mutagenesis of *Beauveria bassiana* (Hypocreales: Clavicipitaceae) yields two hypervirulent isolates with different transcriptomic profiles. Pest Manag. Sci..

[15] Cavalieri D., Di Paola M., Rizzetto L., Tocci N., De Filippo C., Lionetti P., Ardizzoni A., Colombari B., Paulone S., Gut I.G. (2018). Genomic and Phenotypic Variation in Morphogenetic Networks of Two *Candida albicans* Isolates Subtends Their Different Pathogenic Potential. Front. Immunol..

[16] Qiao W., Tang T., Ling F. (2020). Comparative transcriptome analysis of a taxol-producing endophytic fungus, *Aspergillus aculeatinus* Tax-6, and its mutant strain. Sci. Rep..

[17] Huang T.Y., Lee Y.Y., Vidal-Diez de Ulzurrun G., Hsueh Y.P. (2020). Forward genetic screens identified mutants with defects in trap morphogenesis in the nematode-trapping fungus *Arthrobotrys oligospora*. G3.

[18] Brenner S. (1974). The genetics of Caenorhabditis elegans. Genetics.

[19] Dharmendra Kumar D.K., Neelam Maurya N.M., Pintoo Kumar P.K., Harvansh Singh H.S., Addy S.K. (2015). Assessment of germination and carnivorous activities of a nematode-trapping fungus *Arthrobotrys dactyloides* in fungistatic and fungicidal soil environment. Biol. Control.

[20] Persmark L., Nordbring-Hertz B. (1997). Conidial trap formation of nematode-trapping fungi in soil and soil extracts. Fems Microbiol. Ecol..

[21] Chen S., Zhou Y., Chen Y., Gu J. (2018). fastp: An ultra-fast all-in-one FASTQ preprocessor. Bioinformatics.

[22] Jung Y., Han D. (2022). BWA-MEME: BWA-MEM emulated with a machine learning approach. Bioinformatics.

[23] McKenna A., Hanna M., Banks E., Sivachenko A., Cibulskis K., Kernytsky A., Garimella K., Altshuler D., Gabriel S., Daly M. (2010). The Genome Analysis Toolkit: A MapReduce framework for analyzing next-generation DNA sequencing data. Genome Res..

[24] Li H., Handsaker B., Wysoker A., Fennell T., Ruan J., Homer N., Marth G., Abecasis G., Durbin R., 1000 Genome Project Data Processing Subgroup (2009). The Sequence Alignment/Map format and SAMtools. Bioinformatics.

[25] Freed D., Aldana R., Weber J.A., Edwards J.S. (2017). The Sentieon Genomics Tools—A fast and accurate solution to variant calling from next-generation sequence data. bioRxiv.

[26] Wu T., Hu E., Xu S., Chen M., Guo P., Dai Z., Feng T., Zhou L., Tang W., Zhan L.I. (2021). clusterProfiler 4.0: A universal enrichment tool for interpreting omics data. Innovation.

[27] Kim D., Langmead B., Salzberg S.L. (2015). HISAT: A fast spliced aligner with low memory requirements. Nat. Methods.

[28] Pertea M., Pertea G.M., Antonescu C.M., Chang T.C., Mendell J.T., Salzberg S.L. (2015). StringTie enables improved reconstruction of a transcriptome from RNA-seq reads. Nat. Biotechnol..

[29] Dewey C.N. (2011). RSEM: Accurate transcript quantification from RNA-Seq data with or without a reference genome. BMC Bioinform..

[30] Love M.I., Huber W., Anders S. (2014). Moderated estimation of fold change and dispersion for RNA-seq data with DESeq2. Genome Biol..

[31] Zhu Y., Yang X., Bai N., Liu Q., Yang J. (2023). AoRab7A interacts with AoVps35 and AoVps41 to regulate vacuole assembly, trap formation, conidiation, and functions of proteasomes and ribosomes in *Arthrobotrys oligospora*. Microbiol. Res..

[32] Schmidt A.R., Dörfelt H., Perrichot V. (2008). *Palaeoanellus dimorphus* gen. et sp. nov. (Deuteromycotina): A Cretaceous predatory fungus. Am. J. Bot..

[33] Barron G.L. (1981). Predators and Parasites of Microscopic Animals. Biol. Conidial Fungi.

[34] Drechsler C.C. (1933). Morphological diversity among fungi capturing and destroying nematodes. J. Wash. Acad. Sci..

[35] Chen S.A., Lin H.C., Schroeder F.C., Hsueh Y.P. (2021). Prey sensing and response in a nematode-trapping fungus is governed by the MAPK pheromone response pathway. Genetics.

[36] Kuo C.-Y., Chen S.-A., Hsueh Y.-P. (2020). The High Osmolarity Glycerol (HOG) Pathway Functions in Osmosensing, Trap Morphogenesis and Conidiation of the Nematode-Trapping Fungus *Arthrobotrys oligospora*. J. Fungi.

[37] Zhen Z., Xing X., Xie M., Yang L., Yang X., Zheng Y., Chen Y., Ma N., Li Q., Zhang K.Q. (2018). MAP kinase Slt2 orthologs play similar roles in conidiation, trap formation, and pathogenicity in two nematode-trapping fungi. Fungal Genet. Biol..

[38] Zhang W., Hu C., Hussain M., Chen J., Xiang M., Liu X. (2019). Role of Low-Affinity Calcium System Member Fig1 Homologous Proteins in Conidiation and Trap-Formation of Nematode-trapping Fungus *Arthrobotrys oligospora*. Sci. Rep..

[39] McMillan J.N., Sia R.A., Bardes E.S., Lew D.J. (1999). Phosphorylation-independent inhibition of Cdc28p by the tyrosine kinase Swe1p in the morphogenesis checkpoint. Mol. Cell. Biol..

[40] Nakayama N., Kaziro Y., Arai K.I., Matsumoto K. (1988). Role of STE genes in the mating factor signaling pathway mediated by GPA1 in *Saccharomyces cerevisiae*. Mol. Cell. Biol..

[41] Konopka J.B., Jenness D.D., Hartwell L.H. (1988). The C-terminus of the *S. cerevisiae* alpha-pheromone receptor mediates an adaptive response to pheromone. Cell.

[42] Sridhar P.S., Vasquez V., Monteil-Rivera F., Allingham J.S., Loewen M.C. (2023). A peroxidase-derived ligand that induces *Fusarium graminearum* Ste2 receptor-dependent chemotropism. Front. Cell. Infect. Microbiol..

