# Supplementary material for: Genomic and Transcriptomic Analyses Revealed DdSTE2 Play a Role in Constricting Ring Formation in the Nematode-Trapping Fungi Drechslerella dactyloides
Source: Microorganisms. 2024 Oct 30;12(11):2190. doi: 10.3390/microorganisms12112190 (PMC11596859; doi:10.3390/microorganisms12112190)
Supplement: Supplementary file 1 [file microorganisms-12-02190-s001.zip › microorganisms-3235526-supplementary/Figures S1-S9.pdf]

Supplementary materials

Genomic and transcriptomic analyses revealed DdSTE2 play a role in constricting ring formation in the nematode-trapping fungi *Drechslerella dactyloides*

Cheng-Lin Wu[1], Ren-Qiao Wang[1], Jin-Ting Yang[1], Jia-Mei Sun[1], Yan-Rui Xu[1], Jianping Xu[2], Ke-Qin Zhang[1]*, Lian-Ming Liang[1]*

[1] State Key Laboratory for Conservation and Utilization of Bio-Resources in Yunnan, Yunnan University, Kunming, China.

[2] Department of Biology, McMaster University, Hamilton, ON, Canada.

*Correspondence: lianglm@ynu.edu.cn; kqzhang@ynu.edu.cn

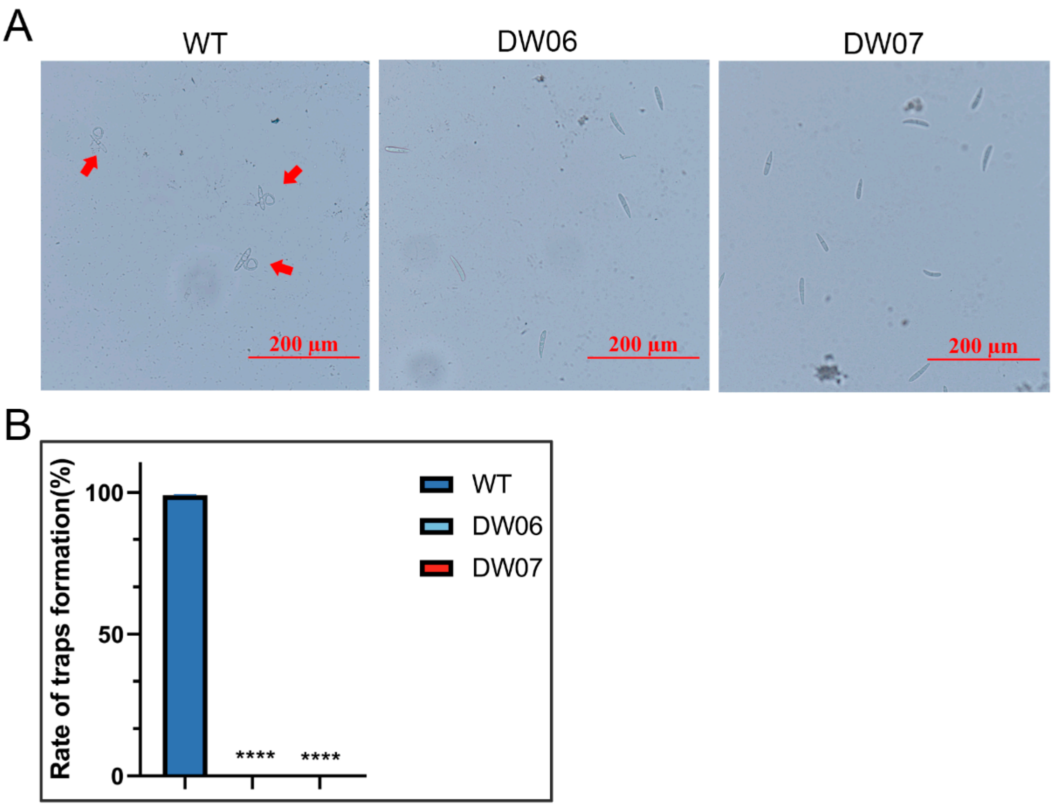

Figure S1 Conidia trap-forming capacity of wild-type (WT) and UV-induced mutants (DW06 and DW07). (A)Conidia traps of WT, DW06 and DW07 were formed after 48 h of soil suspension induction.(B) The proportion of conidia forming traps after 48 h of soil suspension induction.

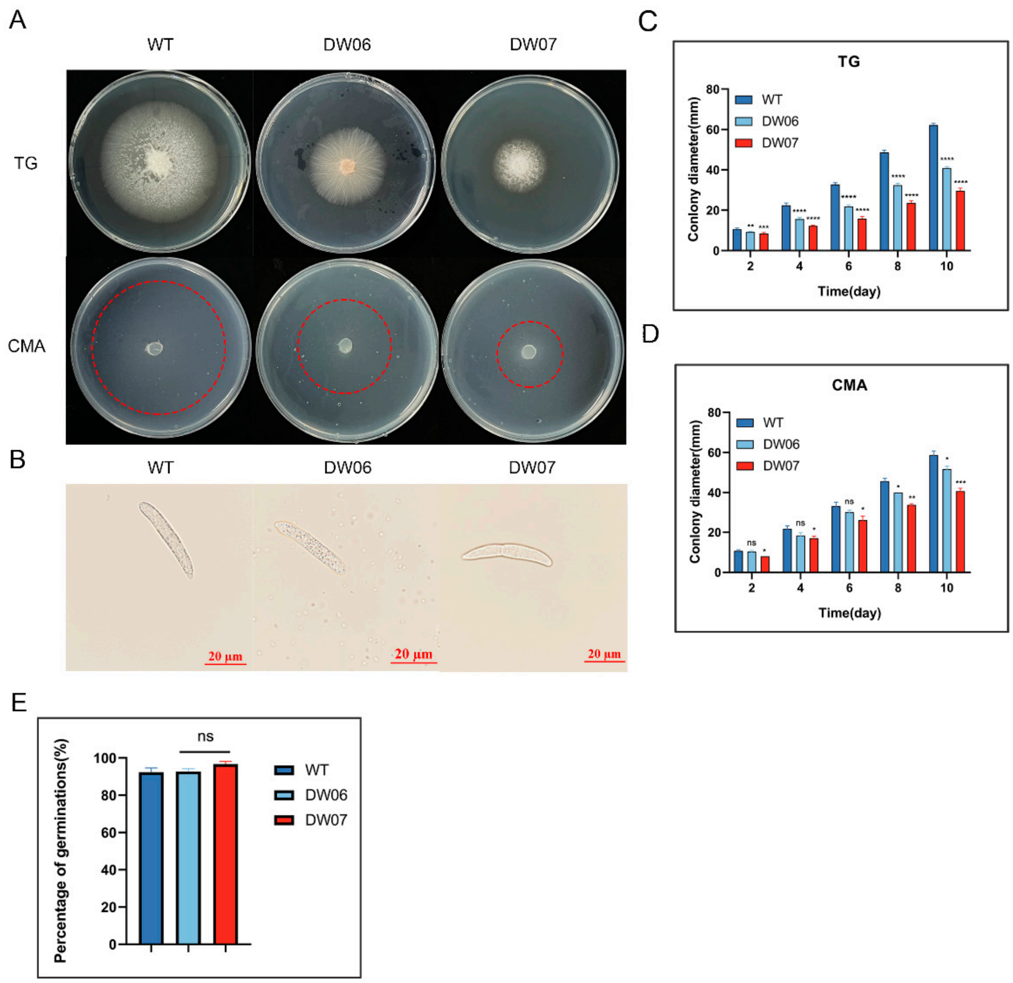

Figure S2 Colony morphology and growth rate on TG and CMA media, and percentage of conidia germination in wild-type (WT) strain and UV-induced mutants (DW06 and DW07). (A) Colony morphology of WT and mutants on TG and CMA media. (B) Microscopic observation of conidia morphology of WT, DW06, and DW07. (C) Colony diameter of WT and mutants on TG medium. Data represent mean ± standard deviation (SD) (n = 5). (D) Colony diameter of WT and mutants on CMA medium. Data represent mean ± standard deviation (SD) (n = 4). (E) Comparison of conidia germination rates of wild-type and UV-induced mutant strains. Data represent mean ± standard deviation (SD) (n = 3). In (C) and (D), two-way ANOVA analysis was conducted. In (E), t-test analysis was performed. *, $P \leq 0.05$; **, $P \leq 0.01$; ***, $P \leq 0.001$; ****, $P \leq 0.0001$; ns, $P > 0.05$.

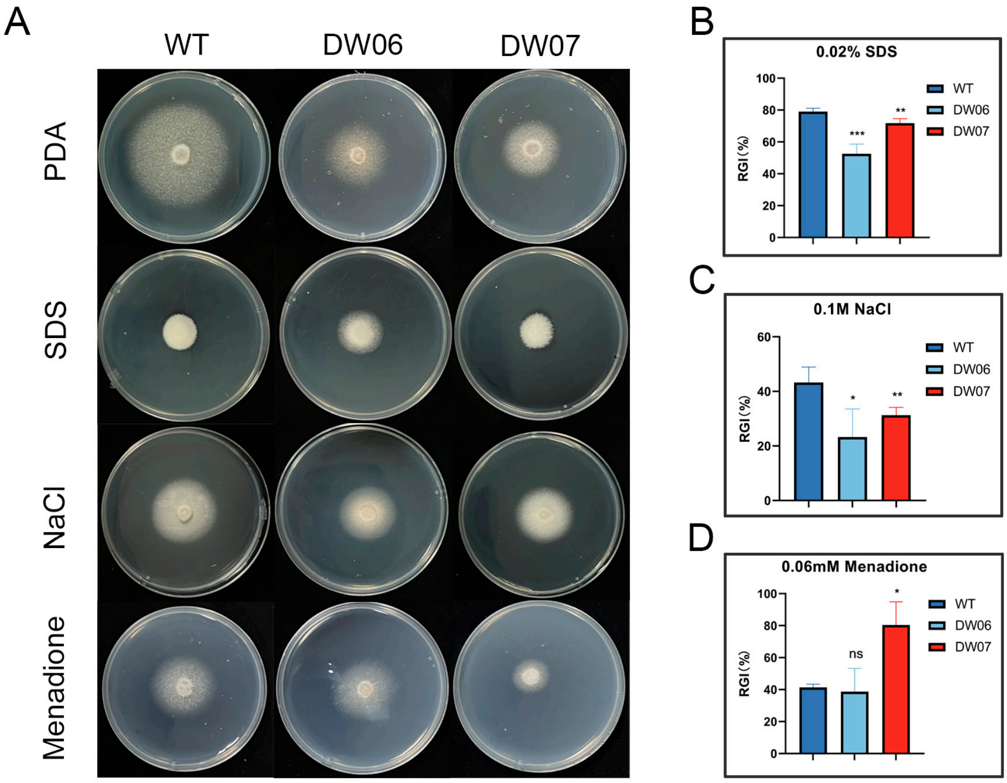

Figure S3 Tolerance of wild-type (WT) and UV-induced mutants (DW06 and DW07) to osmotic, oxidative, and cell wall inhibitory stress. (A) Colony morphology of WT, DW06, and DW07 on PDA medium and PDA medium supplemented with 0.02% SDS, 0.1M NaCl, and 0.06mM Menadione. (B-D) Relative inhibition of growth of WT, DW06, and DW07 on PDA medium supplemented with 0.02% SDS, 0.1M NaCl, and 0.06mM Menadione. Data represent mean ± standard deviation (SD) (n = 4). In (B-D), t-test analysis was conducted. *, $P < 0.05$; **, $P < 0.01$; ***, $P < 0.001$; ****, $P < 0.0001$; ns, not significant.

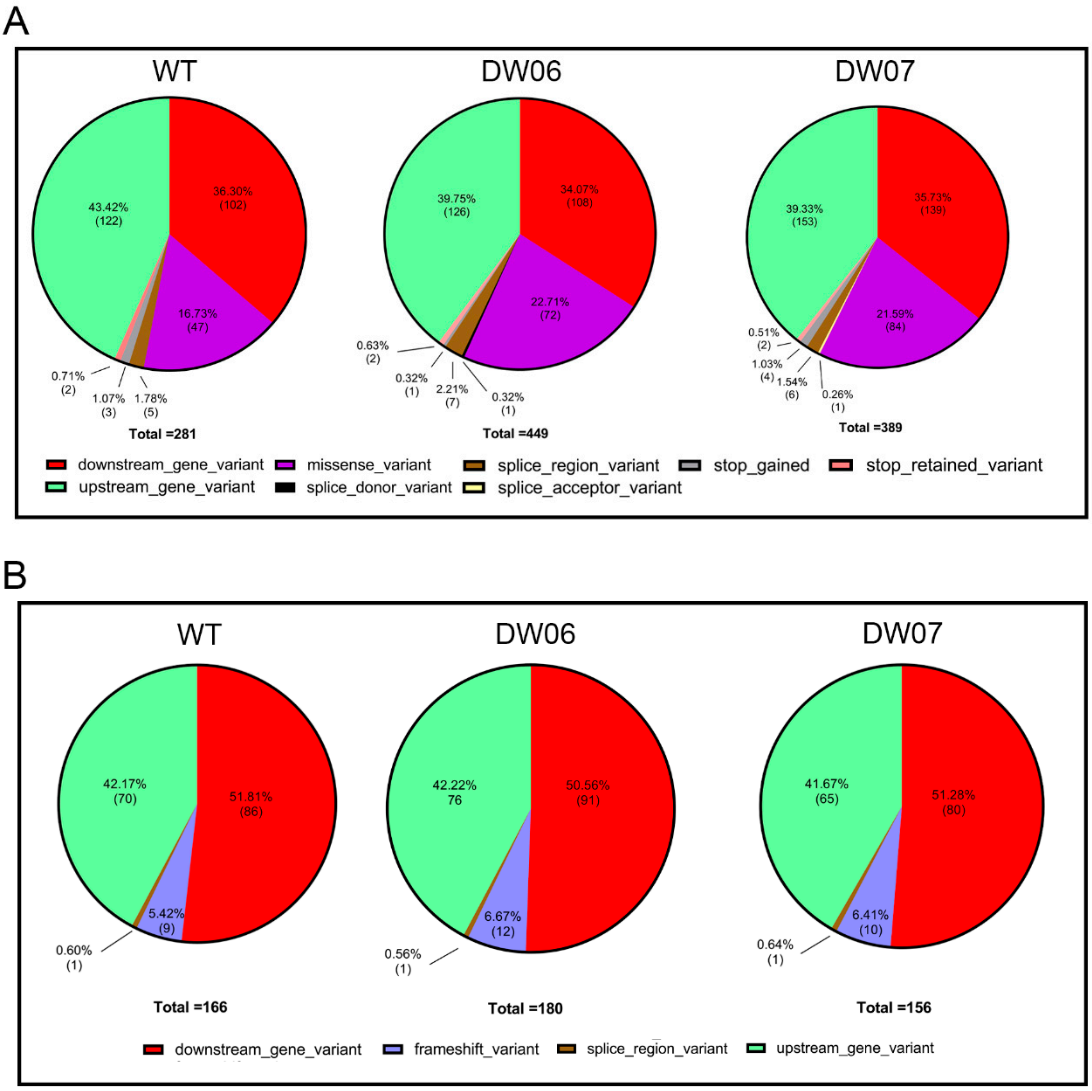

Figure S4 Functional annotation analysis of wild-type(WT) and UV-induced mutants (DW06 and DW07) compared to reference genomic SNPs and InDel.(A) Functional annotation analysis of wild-type(WT) and UV-induced mutants (DW06 and DW07) compared to reference genomic SNPs.(B) Functional annotation analysis of wild-type(WT) and UV-induced mutants (DW06 and DW07) compared to reference genomic InDel.

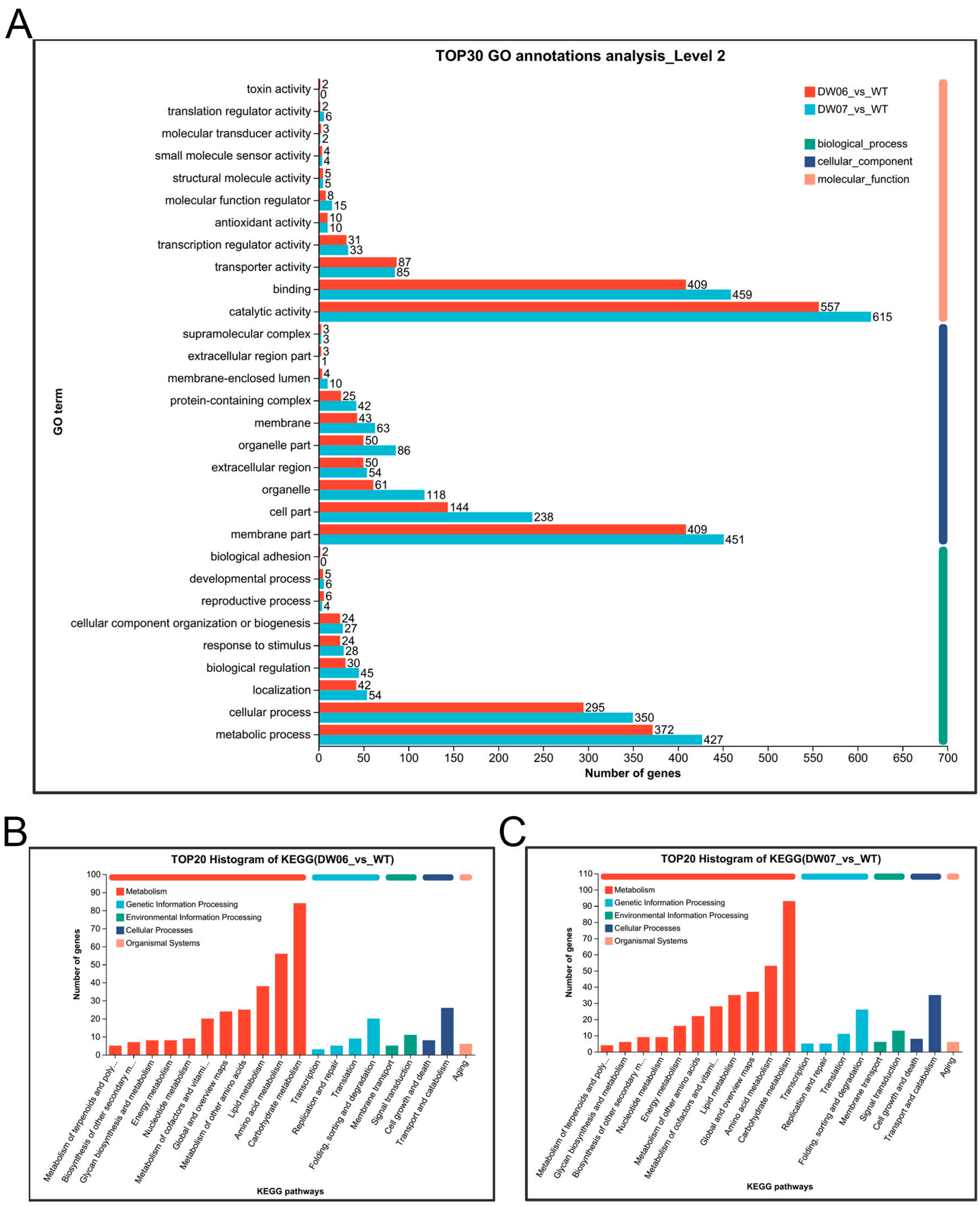

Figure S5 GO and KEGG annotations. (A) Top 30 enriched GO_level_2 annotations in the DW06 vs WT and DW07 vs WT comparison gene sets. (B) and (C) Top 20 enriched KEGG annotations in the DW06 vs WT and DW07 vs WT comparison gene sets.

A

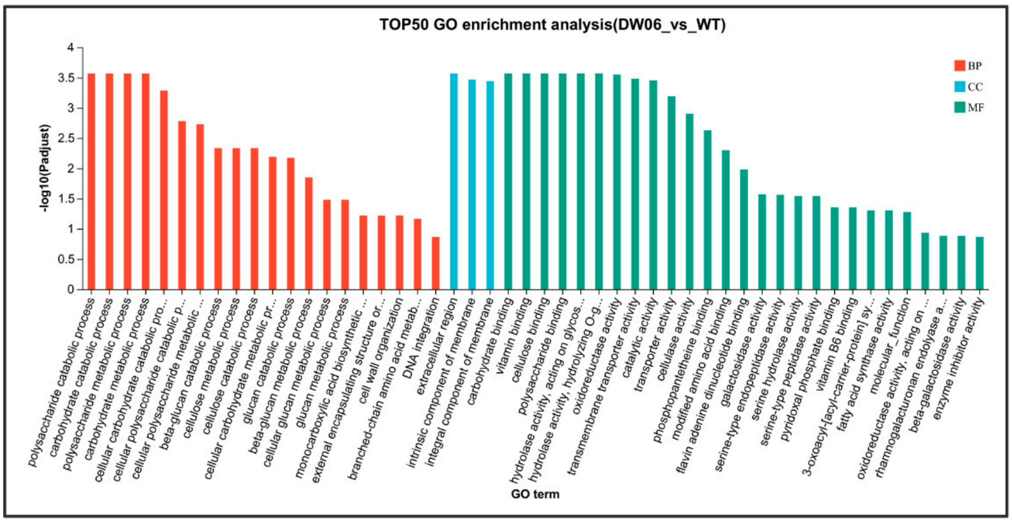

B

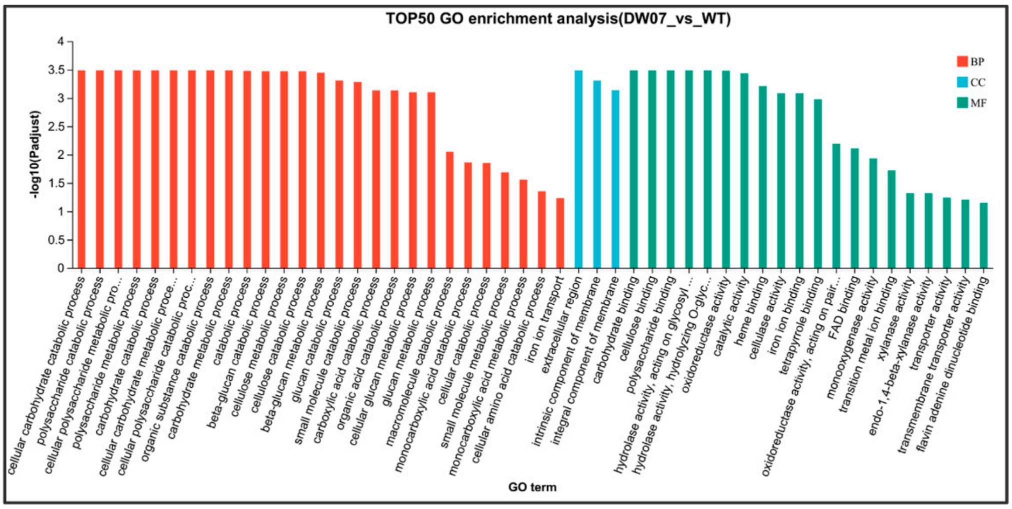

Figure S6 The top 50 most significant GO terms enrichment results for DW06 vs WT gene sets and DW07 vs WT gene sets.

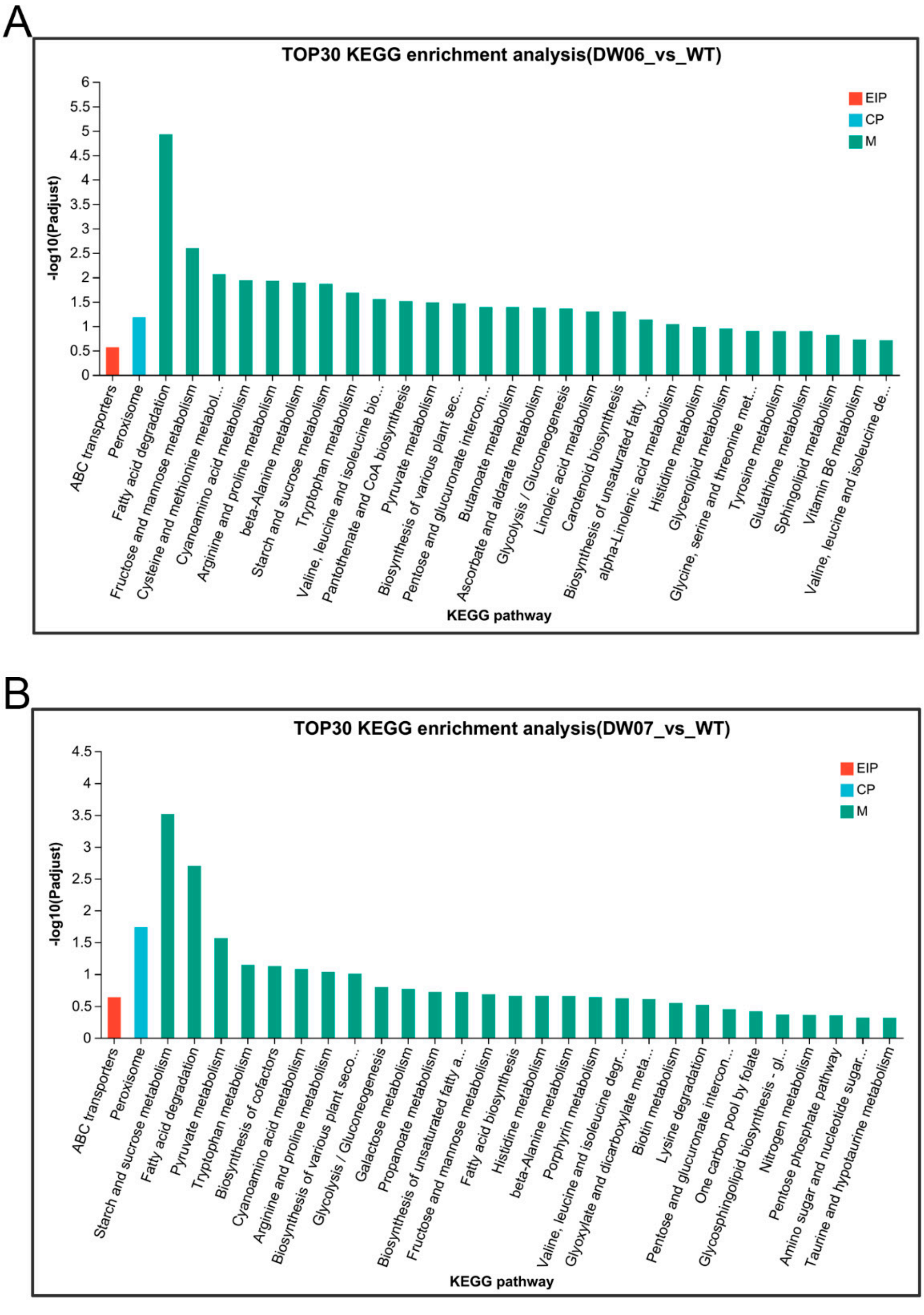

Figure S7 The top 30 most significant KEGG pathways enrichment results for DW06 vs WT gene sets and DW07 vs WT gene sets.

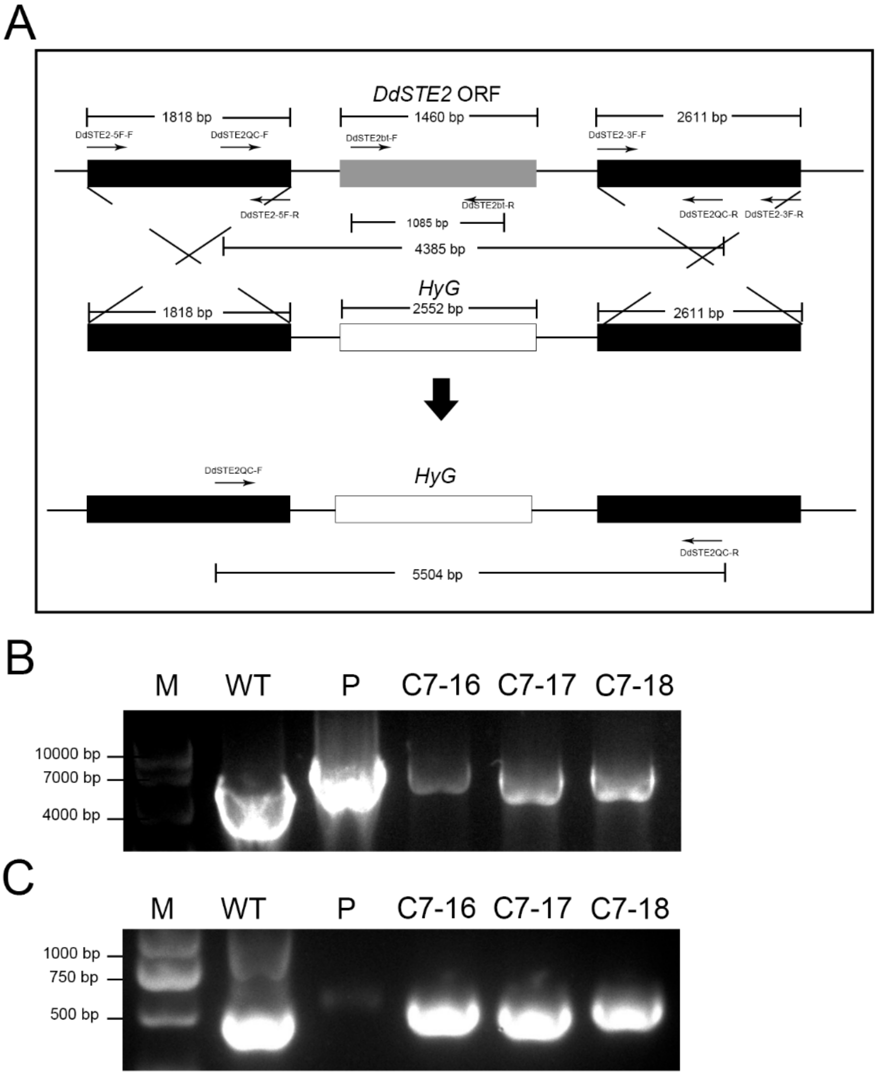

Figure S8 Disruption strategy and verification of *ΔDdaSTE12*. M: Marker; WT: Wild-type genome; P: Knockout the fragment vector plasmid; C7-16: *△DdSTE2* mutants C7-16 genome; C7-17: △DdSTE2 mutants C7-17 genome; C7-18: *△DdSTE2* mutants C7-18 genome.(A) Disruption strategy of *ΔDdSTE2*. The primer locations and expected PCR product lengths are indicated.(B) Verify the presence of *DdSTE2* gene. Tubulin with primers Tubulin-F and Tubulin-R as an internal control.(C) Gene length validation of *△DdSTE2* mutants.

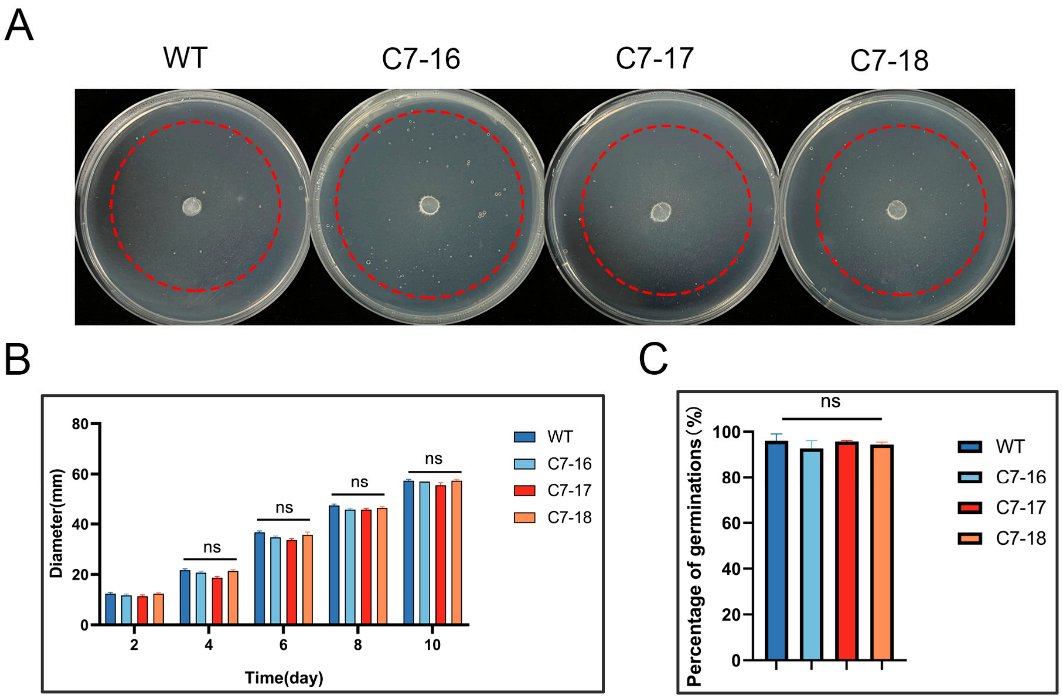

Figure S9 Colony morphology and rowth rate on CMA medium, and percentage of conidia germination in wild-type (WT) strain and *△DdSTE2* mutants (C7-16, C7-17 and C7-18). (A) Colony morphology of WT and *△DdSTE2* mutants on CMA medium. (B) Colony diameter of WT and *△DdSTE2* mutants on CMA medium. Data represent mean ± standard deviation (SD) (n = 4). (C) Comparison of conidia germination rates of wild-type and *△DdSTE2* mutants. Data represent mean ± standard deviation (SD) (n = 3). In (B), two-way ANOVA analysis was conducted. In (C), t-test analysis was performed. *, $P \leq 0.05$; **, $P \leq 0.01$; ***, $P \leq 0.001$; ****, $P \leq 0.0001$; ns, $P > 0.05$.
