# Supplementary material for: Genomic and Transcriptomic Analyses Revealed DdSTE2 Play a Role in Constricting Ring Formation in the Nematode-Trapping Fungi Drechslerella dactyloides
Source: Microorganisms. 2024 Oct 30;12(11):2190. doi: 10.3390/microorganisms12112190 (PMC11596859; doi:10.3390/microorganisms12112190)
Supplement: Supplementary file 1 [file microorganisms-12-02190-s001.zip › microorganisms-3235526-supplementary/Tables S1-S6.pdf]

Table S1. Primers used in qPCR verification

Gene	Primer sequence (5'-3')
β-Tubulin	GAAACTCCACCTCCATCCA
	TCTCGTCCATACCCTCACC
Dda_0210	GGCGAGGCTACCATTTCC
	TCCAGCCGCTTATCAGGT
Dda_3377	AACAATGCGGGCGTCTTT
	GCAACCTTCTGCGTGAGT
Dda_5701	GGTGGTCACGGAAGGGTT
	TAGCCGGCGTATTCCCAATC
Dda_3282	TCCTCCAGGCATCAAAGTG
	CGTCAACCAGCCGTCTATC
Dda_0189	CAGACTGCTGTCGGAACGG
	GAAGAAACGCTGGCTGGAAC
Dda_1334	CTGCCCAGCGTCTTTCAC
	CCTTGCCGCCCTTAGTGA
Dda_2984	CGGTTGACGCTCCTGGTTT
	CGCACGGAGGAACTGTAAGAC
Dda_8119	GACATTCTTGAAATCCCACCC
	CGTAACGCCAGCCTCCTT
Dda_7173	CCGTCTACTGCCTCAACAAG
	AACAGTCCAGCCATTCCCT
Dda_8447	CCCCAAGCAATCCCATACC
	TGGAGACGCCTGCGATAAA
Dda_4244	ATGGACAGGGCATACTCACT
	CAGAATCACCGCCACAGAC
Dda_6033	TTGGCAAGTCGCTGTTCG
	GCTGTCGGCGGTGATGTA
Dda_9344	CCTCCATCAGCCGAGTCAA
	CGAGATACGGACGGCGATA
Dda_7450	CGTCGGCGTTGAGTTTGA
	TCGTCTGCTCCGTTCTGC
Dda_0366	ATTACCGCACGCAGAAGG
	CCAACACCGCCATACACC
Dda_8810	GAGTAGTAGTTGTGGTGAGGGTG
	CCAGGTGGTGGCGTAAAGT
Dda_0739	TGTCACAGCACCAACAAGC
	TTGCCACCGATACCTTCC
Dda_7765	CCCAGGTGAAAGTTGCGTAG
	CGTGGTTCTCGCTGTGATG
Dda_4825	CGACGAATCAGAGGACGAG
	CGACGCCAAGCAGGTTAT

Dda_8468	CGATAAGGATAAGCGAAGGC
	CGAAGAGCACAAGGACGAA

Table S2. Primers used in knockout fragment construction and positive transformant validation

Primer Name	Primer sequence (5'-3')
DdSTE2-5F-F	GCCAGTGAATTCGAGAAGAGGCTGGCAGTAAGAAA
DdSTE2-5F-R	CCAGCCCGGGCCTTGATGAGAATGCGATGAAGGTG
DdSTE2-3F-F	CGGATTTAAATTAATGGAGGTTGAAGGCTGTCG
DdSTE2-3F-R	CATGCTGCAGGTCGATCTCCCACTCAAGCCAATG
DdSTE2bt-F	GGTTCTCGCTGTGATGCTCT
DdSTE2bt-R	TGGTTCGCCTCAGGGTTC
DdSTE2QC-F	ACACCTACCCTCACCACAA
DdSTE2QC-R	TTCGTATCTGTTCACCCACT
Tubulin-F	CTCGCTTGAAGAGCTCCTG
Tubulin-R	ATGCGTACCCTCAAGCTCTC

Table S3. statistical tables of SNP

Sample ID	SNP Number	Transition	Transversion	Ts/Tv	Heterozygosity Number	Homozygosity Number
DW06	739	605	134	4.51	651	385
DW07	875	697	178	3.92	674	371
WT	699	576	123	4.68	678	373

Table S4. statistical tables of InDel

Sample ID	Insertion Number	Deletion Number	Heterozygosity Number	Homozygosity Number
DW06	248	139	275	112
DW07	260	134	281	113
WT	243	142	299	86

Table S5.SNP function annotations

sampleID	WT	DW06	DW07
3_prime_UTR_variant	0	0	0
5_prime_UTR_premature_start_codon_gain_variant	0	0	0
5_prime_UTR_variant	0	0	0
HIGH	3	2	5
LOW	100	110	115
MODERATE	47	72	84
MODIFIER	664	673	799
downstream_gene_variant	102	108	139

intergenic_region	246	240	349
intron_variant	305	319	326
missense_variant	47	72	84
non_coding_transcript	0	0	0
splice_acceptor_variant	0	0	1
splice_donor_variant	0	1	0
splice_region_variant	5	7	6
start_lost	0	0	0
stop_gained	3	1	4
stop_lost	0	0	0
stop_retained_variant	2	2	2
synonymous_variant	96	105	110
upstream_gene_variant	122	126	153

Table S6.InDel function annotations

sampleID	WT	DW06	DW07
3_prime_UTR_truncation	0	0	0
3_prime_UTR_variant	0	0	0
5_prime_UTR_truncation	0	0	0
5_prime_UTR_variant	0	0	0
HIGH	9	12	10
LOW	1	1	1
MODERATE	0	0	0
MODIFIER	382	383	388
bidirectional_gene_fusion	0	0	0
conservative_inframe_deletion	0	0	0
conservative_inframe_insertion	0	0	0
disruptive_inframe_deletion	0	0	0
disruptive_inframe_insertion	0	0	0
downstream_gene_variant	86	91	80
exon_loss_variant	0	0	0
frameshift_variant	9	12	10
gene_fusion	0	0	0
intergenic_region	228	231	225
intragenic_variant	0	0	0
intron_variant	151	148	161
non_coding_transcript_exon_variant	0	0	0
non_coding_transcript_variant	0	0	0
splice_acceptor_variant	0	0	0
splice_donor_variant	0	0	0
splice_region_variant	1	1	1
start_lost	0	0	0

start_retained_variant	0	0	0
stop_gained	0	0	0
stop_lost	0	0	0
stop_retained_variant	0	0	0
upstream_gene_variant	70	76	65
